# Women’s birth place preferences in the United Kingdom: a systematic review and narrative synthesis of the quantitative literature

**DOI:** 10.1186/s12884-016-0998-5

**Published:** 2016-08-08

**Authors:** Jennifer Hollowell, Yangmei Li, Reem Malouf, James Buchanan

**Affiliations:** 1 Policy Research Unit in Maternal Health and Care, National Perinatal Epidemiology Unit, Nuffield Department of Population Health, University of Oxford, Old Road Campus, Headington, Oxford, OX3 7LF UK; 2 Health Economics Research Centre, Nuffield Department of Population Health, University of Oxford, Oxford, UK

**Keywords:** Pregnancy, Systematic review, Midwifery, Place of birth choice, Low-risk pregnancy, Preferences

## Abstract

**Background:**

Current clinical guidelines and national policy in England support offering ‘low risk’ women a choice of birth setting, but despite an increase in provison of midwifery units in England the vast majority of women still give birth in obstetric units and there is uncertainty around how best to configure services. There is therefore a need to better understand women’s birth place preferences. The aim of this review was to summarise the recent quantitative evidence on UK women’s birth place preferences with a focus on identifying the service attributes that ‘low risk’ women prefer and on identifying which attributes women prioritise when choosing their intended maternity unit or birth setting.

**Methods:**

We searched Medline, Embase, PsycINFO, Science Citation Index, Social Science Index, CINAHL and ASSIA to identify quantitative studies published in scientific journals since 1992 and designed to describe and explore women’s preferences in relation to place of birth. We included experimental stated preference studies, surveys and mixed-methods studies containing relevant quantitative data, where participants were ‘low risk’ or ‘unselected’ groups of women with experience of UK maternity services.

**Results:**

We included five experimental stated preference studies and four observational surveys, including a total of 4201 respondents. Most studies were old with only three conducted since 2000. Methodological quality was generally poor. The attributes and preferences most commonly explored related to pain relief, continuity of midwife, involvement/availability of medical staff, ‘homely’ environment/atmosphere, decision-making style, distance/travel time and need for transfer. Service attributes that were almost universally valued by women included local services, being attended by a known midwife and a preference for a degree of control and involvement in decision-making. A substantial proportion of women had a strong preference for care in a hospital setting where medical staff are not necessarily involved in their care, but are readily available.

**Conclusions:**

The majority of women appear to value some service attributes while preferences differ for others. Policy makers, commissioners and service providers might usefully consider how to extend the availability of services that most women value while offering a choice of options that enable women to access services that best fit their needs and preferences.

**Electronic supplementary material:**

The online version of this article (doi:10.1186/s12884-016-0998-5) contains supplementary material, which is available to authorized users.

## Background

The most recent update of the NICE Guideline for Intrapartum Care [1] recommends that healthy women with straightforward pregnancies should be free to choose the birth setting of their choice and that commissioners and providers should ensure that all four birth settings (home, freestanding midwifery unit (FMU), alongside midwifery unit (AMU) and obstetric unit (OU)) are available to all women. Maternity services in the United Kingdom (UK) are provided by the National Health Service (NHS), a tax-funded healthcare system that provides universal access to services that are free at the point of use. Responsibility for the NHS is devolved to the four constituent countries, so policies can differ across the UK. In England, there is a national policy of offering women a choice of birth setting [2, 3]. Policies differ in Scotland, Wales and Northern Ireland but a choice of birth setting is available in all three countries [4–6].

In England, the largest of the four countries of the UK, the provision of midwifery units, particularly AMUs, has increased substantially in recent years [7]. In 2013, 79 % of women in England lived within a 30 min drive of both an OU and a midwifery unit [7] and more recently a national survey of women’s experiences of maternity care found that 41 % of women were offered a choice of giving birth in a midwivery unit and 18 % were offered the option of a home birth [8]. Notwithstanding this, recent data show that the vast majority of women (87 % in 2013) still give birth in an OU [7]; the home birth rate is static at around 2.3 % of births [9]; and although the number of FMUs has increased slightly in recent years, the proportion of women giving birth in FMUs in England is static and remains below 2 % [10]. There is therefore a need for a better understanding of women’s birth place preferences and of the broader factors that influence where women choose to give birth.

The purpose of this systematic review is to summarise the quantitative evidence on UK women’s birth place preferences with a particular focus on identifying the service attributes that women prefer and on identifying which attributes women prioritise when making a choice between different maternity units and different birth settings. The review focuses on evidence relating to the preferences of healthy women with straightforward pregnancies (‘low risk’ women) since this group should be offered a choice of birth setting according to current clinical guideline [1] and national policy [2].

To our knowledge the evidence on women’s birth place preferences and decision making has not previously been systematically synthesised.

## Methods

This paper reports on one component of a broader systematic review which also encompasses the qualitative evidence relating to factors that may affect women’s choice of place of birth, including beliefs, preferences, knowledge and experience. A joint protocol was developed for the present review and the linked qualitative review. Some aspects of the methods reflect the fact that searches were common to the two reviews.

### Eligibility criteria

Eligibility criteria for the present review were as follows:

#### Type of report

- Full primary research reports, published in a scientific journal between January 1992 and February 2015, in English.

#### Topic of research

- Studies designed to describe and explore women’s preferences in relation to place of birth.

#### Research design

- Quantitative studies including experimental stated preference studies, surveys and other quantitative studies designed to describe or explore women’s preferences, and, mixed methods studies that included an eligible quantitative study. For mixed methods studies, eligibility criteria were applied solely to the quantitative component of the study.

#### Study population and setting

Studies conducted in the UK where the study participants were ‘low risk’ or ‘unselected’ groups of women (i.e. women included irrespective of risk status) who had direct experience of UK maternity services, that is women who were either pregnant or had previously given birth in the UK.

We excluded:

- Studies that collected data from other groups such as partners, healthcare professionals or women of childbearing age irrespective of pregnancy history.
- Studies that contained only incidental quantitative data on women’s preferences.
- Studies that reported only descriptive data on women’s reasons for choosing or not choosing a particular maternity unit or setting where the quantitative component of the study was not explicitly designed to describe or explore women’s preferences.

### Search strategy

We searched Medline, Embase, PsycINFO, Science Citation Index, Social Science Index, CINAHL and ASSIA using a search strategy based on the SPIDER tool [11]. For the reasons explained above, the search strategy (see Additional file 1) was deliberately broad and designed to encompass both the quantitative evidence on preferences required for the present review and also the qualitative evidence relating to factors that may affect women’s choice of place of birth, including beliefs, preferences, knowledge and experience. The searches were run in March 2015.

### Study selection

Two reviewers independently screened titles and abstracts followed by double screening of full-text articles where needed. Because this review was conducted as one component of a broader systematic review, the screening was conducted by sequentially applying the criteria applicable to each component of the review, with reviewers working in pairs. At each stage discrepancies were resolved by discussion, with a third reviewer involved as required (see Additional file 1 for further details). We also searched bibliographies of included studies to identify additional eligible studies.

### Quality assessment

We were unable to identify a single critical assessment tool that could be applied across methodologies and found that many of the available tools were unsuitable for assessing surveys. We therefore used a modified version of the Centre for Evidence-Based Managment tool [12] to appraise the included surveys and additionally appraised the experimental stated preference studies using a checklist developed by the International Society for Pharmacoeconomics and Outcomes Research (ISPOR) [13]. Surveys were assessed by YL and RM; stated preference studies were appraised by JB. Eligible studies were included irrespective of quality.

### Data extraction and analysis

Using a data extraction form designed by the authors, JH extracted descriptive data on study context and study objectives, study methods, sample characteristics, sample size, response rate, study period and choices available to study participants, and also wrote a text description summarising the preference-related findings in each report. These data were cross-checked by YL against the full-text articles and any queries regarding the data or interpretation were discussed and resolved. In order to facilitate the production of a narrative summary, findings relating to preferences were coded using a set of keywords e.g. continuity of care, pain relief in labour, decision making, ‘home-like’. These keywords were refined as coding progressed and papers were iteratively recoded where necessary. Eppi-Reviewer 4 software [14] was used for data extraction, coding and data management.

## Results

### Results of the search

**Fig. 1:**
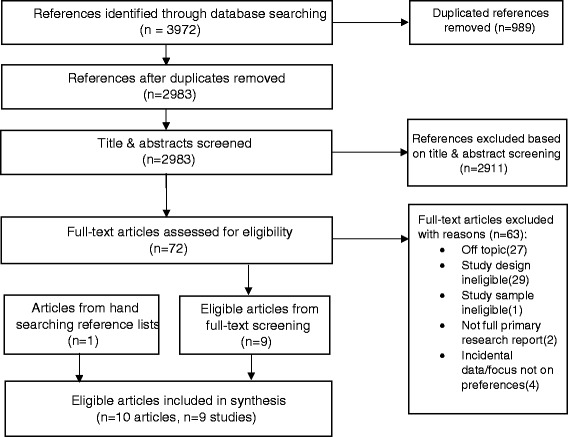
PRISMA Flow chart for study selection process

Our search identified 2983 unique references. Following screening and checking of reference lists of articles eligible for inclusion we identified a total of 10 eligible reports (see Fig. 1 for screening flow chart). These included two pairs of linked papers: the two papers by Hundley [15, 16] reported on different analyses so were included as separate studies while the methodological report by Ratcliffe [17] covered the findings also reported in Longworth [18]. We therefore consider these two as a single study and only included the report by Longworth. The following synthesis is therefore based on nine studies, including 4201 respondents in total.

### Description of included studies

The included studies are described in Table 1 (see Additional file 2 for additional details of study methods).

**Table 1:** Description of included quantitatative studies

Study	Study context/objective	Methods, sample characteristics, response rate and sample size	Study period	Choices compared
Donaldson (1998) [19]	This study was conducted in Aberdeen (Scotland), an area with an OU and an AMU in the same hospital, to assess the feasibility of the use of ‘willingness to pay’ as a measure of women’s strengths of preference for intrapartum care (OU vs. AMU).	Methods: Willingness to pay study designed to evaluate ‘low risk’ women’s preference for type of intrapartum care (OU vs. AMU) at around the time of the booking visit. Questionnaires were mailed to ‘low risk’ women before booking. Sample characteristics: Women at ‘low obstetric risk’. No details reported. Response rate: 75 %, *n* = 113 (only 102 questionnaires (69 %) were used for analysis for various reasons).	May 1994	Hypothetical attributes of OU vs. AMU.
Emslie (1999) [21]	This study was conducted to explore women’s preferences and experiences following the opening of an FMU in the study area (Peterhead near Aberdeen in Scotland). Women in this area had four choices: home birth, FMU and both OU and AMU available approximately 35 miles away (in Aberdeen). A DOMINO (Domiciliary in and out) delivery service was also available to women registered with the FMU. The FMU was based in the Peterhead Community Hospital. The largest general practice is located in Peterhead with two rural practices in the surrounding area.	Methods: Questionnaire survey mailed to women in the FMU’s catchment area at around 14 weeks gestation, at 36 weeks gestation and 6 weeks postnatally. This survey was one component of a mixed methods study. Sample characteristics: Over half (59 %) of respondents (*n* = 254) were registered with the main GP practice in the FMU catchment area; 41 % of women were nulliparous; 70 % were aged under 29 years and 28 % were under 24 years of age. Response rate: 77 % for 14 week survey, *n* = 254. Of these 83 % responded to 36 week survey, *n* = 210.	January to December 1995	Study focuses on FMU vs. hospital (OU/AMU) choices made by women in the catchment area of a newly opened FMU.
Hundley (2001) [16]	Pilot study to explore feasibility of using discrete choice experiment to assess women’s preferences for aspects of intrapartum care. The study was conducted in three areas in Grampian, Scotland where different models of care were available. Linked study: Hundley (2004).	Methods: Discrete choice experiment. Data were collected by postal questionnaire from women recruited at booking. Sample characteristics: Of the 301 ‘low risk’ respondents, the mean age was 28; 55 % were nulliparous; the vast majority (91 %) were married or cohabiting. The women were more socioeconomically advantaged than the national population. **Response rate:** Estimated response rate was 40 %, *n* = 301.	January to November 1999	Study evaluates preferences for different service attributes.
Hundley (2004) [15]	This study was conducted to investigate the effect of service provision on consumer preferences, in particular, whether women who have access to systems of care which offer particular attributes value these attributes more highly than women for whom the attributes are not a realistic option. Three groups of ‘low risk’ women participated from areas with different services available (OU/AMU, FMU and OU/AMU without an epidural service). The areas also differed in the degree of continuity of carer offered. For primary report see Hundley (2001).	Methods: Discrete choice experiment. Data were collected by postal questionnaire from women recruited at booking. Sample characteristics: See Hundley (2001) for characteristics of the overall sample. ‘Low risk’ women in the three study groups were similar, but there were more nulliparous women in the Aberdeen (OU/AMU) group and women in the Elgin (OU/AMU without epidural service) group were less deprived. The Peterhead and Elgin groups were relatively small (*n* = 48 and *n* = 60) compared to the Aberdeen group (*n* = 193). Response rate: Estimated response rate overall was 40 %. Response rate varied by area (33 %–44 %), *n* = 301 (193 from the Aberdeen group, 48 from the Peterhead Group and 60 from the Elgin group).	January to November 1999	Preferences for particular service attributes in women with access to: OU/AMU vs. FMU ~30 miles from OU/AMU vs. OU/AMU without an epidural service.
Lavender (2005) [22]	This project was commissioned by the Department of Health (UK) to inform the Children’s National Service Framework. The aim was to identify models of maternity care which provide a safe, equitable and sustainable service that meets the needs of the current and future population and offers choice to women.	Methods: Questionnaire survey of pregnant women in a purposive sample of 12 maternity units in England. Units were included that offered different birth settings (home, FMU, AMU and OU) and varied in size (50 births to 6000 births). This survey was one component of a mixed methods study. Sample characteristics: Half (51 %) of the 2071 questionnaires returned were from district general hospitals (presumed to be OUs), 38 % were from university hospitals incorporating midwife-led units (presumed to be OU/AMUs) and 11 % were from FMUs. The mean age of participants was 29 and the mean gestational age was 29 weeks. Just over half (54 %) were multigravid with most having given birth to one child previously (46 %); 84 % were ‘white-European’ and 90 % had English as a first language; approximately 15 % (*n* = 303) were classified as being from ‘ethnic minority groups’. Response rate: Overall 71 %, with unit response rates varying from 59 to 85 %. *n* = 2071.	January to March 2002	Preferences for a range of service attributes.
Pitchforth (2008) [20]	A discrete choice experiment to evaluate preferences for key attributes of intrapartum care in women living in remote rural areas in Scotland served by FMUs and small consultant units without neonatal facilities.	Methods: Discrete choice experiment. Sample characteristics: The mean age of respondents was 30 years, 43 % women had delivered their first baby. Response rate: 62 %, *n* = 877 (including 22 of whom returned blank questionnaires).	April 2004 to January 2005	Preference for hypothetical attributes of midwifery-led vs. consultant care
Rennie (1998) [23]	A pilot study to identify women’s preferences for aspects of intrapartum care and to evaluate whether they differ in the postnatal period compared with late pregnancy.	Methods: A questionnaire survey of pregnant women at around 34 weeks gestation, with a follow-up questionnaire 10 days after the birth. Sample characteristics: Despite stratified sampling there was a preponderance of nulliparous women (65 %); 81 % of participants were married and two thirds (66 %) were owner occupiers. Most (70 %) were planning to attend antenatal education. The mean age of respondents was 27. Response rate: 96 % for the 34 week survey (*n* = 207); 86 % of respondents also completed the postnatal questionnaire (*n* = 185).	February to March 1996	Study focuses on service attributes preferred antenatally vs. postnatally.
Rogers (2011) [24]	This study was conducted to evaluate the viability of converting an AMU in outer London to an FMU following the planned closure of the OU in the hospital. The study focused on whether users of the existing AMU would choose the new FMU or would look for an alternative.	Methods: A questionnaire survey conducted amongst a cross-sectional sample of ‘AMU users’: women who were either booked, considering booking or who had given birth at the AMU situated in a hospital where a relocation of the OU was planned. Sample characteristics: The majority of study participants were pregnant (89 %) and the remaining 11 % had just had a baby. Sixty percent of participants were nulliparous. Response rate: 53 %, *n* = 121.	October 2009	AMU vs. FMU

Note that the for some studies, the calculation of response rates varied between reports. In these instances we directly quote the response rate reported by the authors

Information on preferences was elicited in various ways. Five studies used discrete choice or other experimental stated preference methods [15, 16, 18–20]; five asked women to rate the aspects of maternity services that were important to them [15, 16, 21–23]. One study reported women’s reasons for choosing a specific unit or setting [24]; and one study asked women to state what factors had affected their booking decision [21].

Five studies [15, 16, 19, 21, 23] were conducted in the same region in and around Aberdeen in Scotland (Grampian). Services in this area included an OU and AMU in Aberdeen, an FMU around 35 miles away and an OU without an epidural service approximately 65 miles from the main OU. Two of these studies used samples of women booking at the main OU/AMU [19, 23]; one study recruited women resident in the catchment area of the FMU [21]; and the two ‘linked’ studies by Hundley [15, 16] recruited ‘low risk’ women booking in three units (OU/AMU, FMU and OU without an epidural service).

One further Scottish study was conducted in remote and rural areas in the North of Scotland where services were provided by small community hospitals (<300 births per year) with a mix of OUs (some without neonatal services) and FMUs [20].

Two studies were conducted in London [18, 24]. In one of these, the evaluation was conducted in an area that was considering shutting its OU and converting the local AMU to an FMU [24] and the study was designed to evaluate whether current AMU users would consider birth in the unit if it became an FMU. The other was a stated preference study conducted in areas with high home birth rates, comparing preferences in women booked for a home birth with ‘low risk’ women booked for a hospital birth [18].

Only one study was carried out in a national sample [22]. This recruited a cross-sectional sample of women from a purposive sample of maternity units selected to provide socioeconomic, ethnic and urban/rural diversity and a mixture of available birth settings (home, FMU, AMU, OU) across England.

Most of the studies were relatively old: only three of the studies [20, 22, 24] had collected data since 2000. The most recent included study [24] was carried out in 2009.

### Methodological quality of included studies

The quality of the surveys was generally fairly low (Additional file 3). Most of the surveys were conducted in single units or small geographical areas. The exception was the survey by Lavender [22], which collected data from a nationally representative sample of units in England. Additionally many of the surveys had low response rates and most of the surveys were small and did not report confidence intervals. Thus many of the descriptive findings reported in these studies have a high risk of bias, estimates of the prevalence of particular preferences are based on small potentially unrepresentative samples, and the generalisability of findings is uncertain.

The five stated preference studies were found to be of mixed but generally average quality (Additional file 4). Almost all appropriately justified the sampling strategy that was used, and study limitations, generalisability and implications were generally adequately discussed. However, several quality criteria were met by very few studies. No studies justified the number of attributes or profiles in each choice task, only one study partially described the study data collection instrument and methods [19] and only one study partially evaluated the properties of the experimental design (e.g. efficiency score, cognitive difficulty) [16]. Other general weaknesses included a lack of justification for attribute selection, limitations relating to experimental design or mode of administration and little consideration of the quality of responses.

### Women’s preferences and service attributes influencing choice

The attributes and preferences most commonly explored related to pain relief (including availability of a birthing pool) [15, 16, 18, 21–23], continuity of midwife [15, 16, 18, 21–23], involvement/availability of medical staff [15, 16, 21, 22, 24], ‘homely’ environment/atmosphere [15, 16, 18, 21, 24], decision-making style [15, 16, 18, 21, 23]. Other factors investigated included distance/travel time [20–22, 24] and need for transfer [18, 22, 24]. One study explored women’s preferences for care in a labour ward vs. a midwifery unit [19] and one explored preferences for midwife-managed vs. consultant-led ‘staffing’ [20]. Pitchforth’s study also dealt with methods of pain relief and involvement of medical staff but these attributes were varied in tandem in the discrete choice experiment to ensure that respondents realised that an epidural was only available with consultant-led care [20]. Relevant findings from this study are therefore only presented under the ‘Distance’ and ‘Obstetric unit vs. midwifey unit’ sections below.

Table 2 lists the attributes and preferences evaluated in each of the studies. Findings relating to specific preferences are summarised below and are also tabulated in Additional file 5.

**Table 2:** Maternity service attributes used to assess preferences in the included studies

Study & method	Preferences evaluated
Donaldson (1998) [19] *Willingness to pay*	Labour ward vs. midwives unit
	Labour ward characterised as:
	- Doctors more likely to be involved in decision-making; midwives involved but women will not see the same midwife all the time; Electronic fetal monitoring; because of monitoring/other reasons 1 in 2 women have limitations on movement during labour; 1 in 12 women try alternative positions for delivery; 1 in 5 have an epidural; 1 in 3 have episiotomy
	Midwives unit characterised as:
	- Decisions made by women and midwives; most care from one midwife; traditional fetal monitoring, transfer to labour ward needed if continuous monitoring required; 1 in 4 women transferred to labour ward for electronic monitoring; because of monitoring/other reasons 1 in 3 have limitations on movement during labour; 1 in 8 try alternative positions for delivery; all types of pain relief available but transfer to labour ward required for epidural; 1 in 7 have an epidural; 1 in 4 have episiotomy
Emslie (1999) [21] *Questionnaire survey - longitudinal follow-up*	Features of place of birth rated by women at 14 and 36 weeks (selected list – not all reported)
	- Quiet atmosphere - Baby with you at all times - Availability of specialist facilities - Convenience for visitors - Choices in pain relief - Choices in delivery
	Aspects of labour management rated by women (at 36 weeks):
	- Partner being there - Availability of specialist staff/equipment - Being kept informed - Being involved in decisions - Time alone with partner - Choice of pain relief - Freedom to choose different positions - Handed baby immediately - Cared for by known staff - Not being left alone - Homely atmosphere - Cared for by named midwife - Being introduced to people - Provision of music/TV
Hundley (2004) [15], Hundley (2001) [16] *Discrete choice experiment*	Continuity (midwife):
	- Meet midwife antenatally, same midwife present throughout labour/birth vs. meet team of midwives antenatally, one present throughout labour/birth vs. previously unknown midwife but present throughout labour/birth vs. midwives working shifts may change during labour/birth
	Pain relief:
	- All methods except epidural vs. all methods available but epidural requires transfer vs. all methods available.
	Fetal monitoring:
	- Continuous, movement may be restricted during labour vs. intermittent unless complications develop, then continuous if required
	Appearance of room:
	- Homely vs. clinical appearance
	Medical staff:
	- Involved in care vs. only involved if complications develop
	Decision-making:
	- Staff make decisions vs. staff make decisions but keep woman informed vs. staff discuss things with women before deciding vs. staff give woman assessment, woman in control of decisions
Lavender (2005) [22] *Questionnaire survey*	Women were asked to state their level of agreement/disagreement with the following:
	- It is not important for me to have my baby in the same place as I receive antenatal care - It is important that my antenatal appointments are at a location close to where I live - I would be willing to travel if it meant I would receive higher quality care for my baby and me around the time of birth - It is important to me that a midwife helps me to give birth to my baby even if complications develop - I would feel unsafe if a specially trained doctor was not immediately available when I am in labour - It is not important to me that a midwife I know helps me to give birth to my baby - It is important to me to that [sic] a special care baby unit is in the same place that I give birth - It is important to me to be able to have an epidural at any time of day or night - It is important to me that a pool is available for my labour/birth - I want to be looked after by midwives and not have doctors involved - I would not want to transfer to a hospital a few miles away if my baby or I develop a problem
Longworth (2001) [18] *Conjoint analysis*	Continuity:
	- Have not met midwives prior to labour vs. have met midwives but don’t know them well vs. know midwives well
	Location:
	- Labour ward vs. maternity unit with a home-like environment vs. home
	Pain relief:
	- Gas & air/breathing only, no epidural, no birthing pool vs. gas & air and birthing pool, no epidural vs. all options including epidural
	Decision-making during labour and delivery:
	- Midwives and doctors will decide vs. decisions will be made jointly following discussion vs. woman will make own decisions
	Probability of transfer to another hospital during labour:
	- No need for transfer if problems develop vs. low probability of transfer vs. high probability of transfer
Pitchforth (2008) [20] *Discrete choice experiment*	Model of care:
	- Consultant-led vs. midwife-managed care - Pain relief: all methods available vs. no epidural^a^
	Distance (‘time travelled’):
	- Zero (home birth) vs. 30 mins vs. 60 mins vs. 90 mins vs. 120 mins
Rennie (1998) [23] *Questionnaire survey*	Aspects of intrapartum care rated by study participants:
	- Birth companion - Known midwife - In control - Few interventions - Able to do what you want - Same midwife in labour - Not to lose control of behaviour - Preferences and wishes followed - Attendance of midwife: - all the time vs. easy access vs. present only when I say - Information: - constant flow vs. staff to decide vs. only when asked for - Option for pain relief - pain-free with drugs vs. minimum drugs vs. drug free labour/other - Decision-making in labour: - staff decides vs. reach decision together vs. woman decides
Rogers (2011) [24] *Questionnaire survey*	Women who would use the local AMU when it becomes a stand-alone unit (FMU) were asked to select reasons for their choice:
	- Easy to get to - Physical environment - Previous bad experience - Previous good experience - Can use water in labour and for birth - Wants natural childbirth - Homely/small - Family can be involved - Other Women who would not use the local AMU when it becomes a stand-alone unit were asked to select reasons for their choice:
	- Difficult to get to - Want an epidural - Feel safer - Previous bad experience - Previous good experience - Physical environment - Pressure from partner/family/friends - Would prefer a midwife-led unit on the same site as the hospital labour ward - Concern about transfer

^a^Note: In Pitchforth’s study, ‘pain relief’ was primarily included to ensure that respondents realised that an epidural was only available with consultant-led care. As such, the levels for this attribute varied in tandem with the levels of the ‘Staff involved’ attribute: the only options that respondents saw were either ‘Midwife-managed care’ and ‘No epidural available’ or ‘Consultant-led care’ and ‘All methods of pain relief’

### Methods of pain relief, including availability of birthing pool

In Hundley’s primary study, 84 % of respondents indicated a preference for having all methods of pain relief available and this appeared to be the second most important attribute to participants (after style of decision-making) [16]. However, further analysis [15] found that ‘pain relief’ did not impact on the preferences of women who lived in areas where the local maternity unit (FMU or OU) did not have an epidural service. The authors comment that their findings are consistent with an ‘endowment effect’, that is, expectations influence preferences [15].

Longworth’s study found that women with a dominant preference for hospital birth exhibited a significant preference for access to all forms of pain relief, whereas (as might be expected) this was not important to women with a dominant preference for home birth. Pain relief options did not appear to be of importance to ‘traders’ who were potentially willing to switch setting in order to access services better meeting their preferences [18].

In Lavender’s national survey, half of respondents agreed or strongly agreed with the statement “It is important to me to be able to have an epidural at any time of day or night”, although the authors noted that this did not necessarily mean that they were intending to have one. Availability of a birthing pool elicited a more uncertain response: 46 % of respondents neither agreed nor disagreed with the statement “It is important to me that a pool is available for my labour/birth”. Around a quarter agreed or strongly agreed with this statement [22].

In Emslie’s study, women living in an FMU catchment area rated the importance of ‘choices in pain relief’ fairly highly, with importance increasing in later pregnancy (81 % considered ‘choices in pain relief’ important at 14 weeks increasing to 95 % at 36 weeks). However, more than half of the respondents were booked to give birth in an FMU and it should be noted that the responses related to ‘choices in pain relief’ and not necessarily to the availability of all options or of epidural. The authors noted that “a sizeable percentage of women would have liked to have known more [about pain relief], especially about natural methods such as massage, breathing, and the role of different positions” ([21], p203).

In Rennie’s study of women booked for birth in a hospital with an OU and AMU, most women (69 %) rated ‘minimum drugs’ as quite important or very important at 34 weeks with only 14 % rating ‘pain free with drugs’ as quite important or very important and 11 % rating ‘drug free labour/other’ as quite important or very important. When asked in the postnatal period, almost all women (95 %) said that ‘effective pain relief’ in labour was quite important or very important [23].

### Continuity of midwife

Two aspects of continuity were explored: first, preferences relating to being attended in labour by a known midwife; and second preferences relating to having the same midwife throughout labour.

In Hundley’s primary study [16], ‘continuity of midwife’ was considered an important attribute by the vast majority of women (95 % considered this quite important or very important), and the majority of women stated a preference for having a known midwife for labour and the same midwife throughout labour and delivery: 69 % chose the option ‘you meet the midwife during your pregnancy and the same midwife is present throughout labour and delivery’ and 23 % expressed a preference for ‘you meet a team of midwives during pregnancy, one of whom is present throughout labour and delivery’. The discrete choice regression analyses confirmed that women tended to prefer scenarios with more continuity of midwife. However, when asked to state which was the most important attribute if they could only be certain of getting one of their choices, ‘midwife’ was considered the preferred attribute by only 17 % of study respondents (after ‘decision-making’ (40 %) and ‘pain relief’ (23 %)).

Further analysis [15] that explored whether women’s preferences were influenced by the services that women had available in their local areas found that in the study area with least continuity available, women were significantly less likely to prefer the option of labour care from a midwife that they had met during pregnancy (52 % vs. 72–75 % in other areas). This is consistent with an ‘endowment effect’, that is expectations influence preferences.

In Longworth’s study women with a dominant preference for hospital birth and women with a dominant preference for home birth both had a significant preference for higher levels of continuity of carer. Amongst ‘traders’ - women prepared to switch setting according to the services and attributes available - continuity of midwife was the only attribute that significantly influenced which setting the woman chose, with higher levels of continuity being preferred [18].

However, in Lavender’s national survey, a statement regarding the importance of care by a ‘midwife I know’ for the baby’s birth did not elicit strong responses: few strongly agreed or strongly disagreed and respondents were fairly equally divided between agreeing, disagreeing and neither agreeing nor disagreeing [22]. Emslie’s study also found that being cared for by a ‘named midwife’ in labour was rated as important by only 18 % of women and being ‘cared for by known staff’ was considered important by 28 % of respondents at 36 weeks [21].

Rennie’s study found that the importance of having a ‘known midwife’ differed antenatally and postnatally. Antenatally, around half of study participants rated having a ‘known midwife’ as quite or very important and 39 % didn’t mind. Postnatally, the proportion of women considering this important fell, with only 29 % of women considering this important, almost half saying they didn’t mind, and 22 % saying it wasn’t important. However, participants attached a higher importance to having the ‘same midwife in labour’ with 69 and 66 % respectively saying this was quite or very important antenatallly and postnatally. With regard to access to a midwife during labour, ‘easy access’ rather than ‘all the time’ or ‘only when I say’ appeared to be the preferred option antenatally. Postnatally, 74 % of women thought that ‘constant attendance of the midwife (during labour)’ was important [23].

### Medical staff involvement/availability of specialist clinical services

Hundley’s primary study explored women’s preferences for medical staff involvement (involved in care vs. only involved if complications develop). When asked to state a preference, two thirds of participants (67 %) said that they preferred to have ‘medical staff (doctors) only involved if required (i.e., a complication occurs)’ and when asked to state what was the most important attribute if they could only be certain of getting one of their choices, only 13 % chose the ‘medical staff’ attribute. However, the discrete choice regression analysis findings indicated that women were more likely to prefer maternity units that offered routine involvement of medical staff [16]. Hundley’s further analysis indicated that women’s preferences for medical staff involvement did not appear to be affected by the services available to them [15].

In Lavender’s national survey, 62 % of women agreed or strongly agreed with the statement “I would feel unsafe if a specially trained doctor was not immediately available when I am in labour”; while 20 % agreed or strongly agreed with the statement “I want to be looked after by midwives and not have doctors involved” [22].

In Emslie’s study, ‘availability of specialist staff/equipment’ was considered important by 65 % of women at 36 weeks, with only ‘partner being there’ considered more important [21].

In Rogers’ study which investigated whether AMU-users would choose to have their baby in the unit if it became an FMU, 57 % of nulliparous women and 71 % of multiparous women said that they would choose to have the baby in the unit if the OU closed and the AMU became an FMU. Amongst women (*n* = 21) who said that they would not choose the unit if it became an FMU, 81 % said that they would prefer a midwife-led unit on the same site as an OU and 67 % stated that they would ‘feel safer’ elsewhere [24].

### ‘Homely’ environment and atmosphere

In Hundley’s primary study, 92 % of women expressed a preference for a unit that had a ‘homely or homelike appearance’, rather than a ‘clinical appearance’. The discrete choice analysis confirmed that women tended to choose options that provided a ‘homely or homelike’ room. However, when asked to state what was the most important attribute if they could only be certain of getting one of their choices (see Table 2 for list), less than 2 % considered the ‘appearance of room’ to be the most important attribute [16]. Further analysis of the data did not suggest that women’s preferences for a homely room were affected by the characteristics of the local services available to them [15].

Longworth’s study explored women’s preferences for ‘location’ options including ‘maternity unit with a home-like environment’, ‘hospital labour ward’ and ‘home’. The findings did not strongly suggest that a ‘homely environment’ was important to ‘traders’ who might be prepared to switch setting in order to access services that better met their preferences [18].

In Rogers’ study, the majority of AMU-users said that they would choose to have their baby in the unit if it became an FMU, with ‘homely/small’ being one of the most commonly cited reasons for using the FMU (cited by 67 % of those who said they would choose the FMU) [24].

In Emslie’s study, a ‘homely atmosphere’ was stated to be important by 18 % of women while a ‘quiet atmosphere’ was considered important by the vast majority of women [21].

### Style of decision-making

In Hundley’s primary study, the vast majority of women expressed a preference for being involved in decision-making: 48 % preferred the option ‘the staff give you their assessment, but you are in control of the decision’ and 42 % preferred ‘the staff discuss things with you before reaching a decision’. When asked to state which was the most important attribute if they could only be certain of getting one of their choices, decision-making was the most frequently chosen attribute, with 40 % of women selecting this as the most important [16]. Further analysis indicated that decision-making preference did not appear to be affected by the services available to the women [15].

Women in Longworth’s study also expressed a preference for more autonomy in decision-making [18].

Rennie’s study found that antenatally, around two thirds of women considered reaching a decision together with health care staff to be ‘very’ or ‘quite’ important, with other respondents almost equally split between ‘staff decides’ and ‘woman decides’ [23].

In Emslie’s study, 53 % of women stated at 36 weeks that ‘being involved in decisions’ was important to them [21].

### Distance

In Pitchforth’s study, conducted in women living in remote and rural areas in Northern Scotland, women preferred to deliver in maternity units rather than at home and preferred shorter travel times to access intrapartum care. However, the analysis also revealed that women were prepared to travel up to 133 min from home to receive consultant (OU) care and that they would travel 16 min further to receive consultant-led care vs. alternatives. Remoteness clearly influenced women’s willingness to travel with women living in particularly remote areas willing to travel further [20].

In Lavender’s national survey, one of the statements that elicited the strongest agreement related to distance: 68 % agreed or strongly agreed with the statement “I would be willing to travel if it meant I would receive higher quality care for my baby and me around the time of birth” [22].

In Rogers’ study investigating whether AMU-users would choose to have their baby in the unit if it became an FMU, the majority said that they would, with ‘easy to get to’ being the fourth most commonly cited reason for using the FMU (cited by 54 % of those who said they would choose the FMU) [24].

In Emslie’s study, ‘distance from home’ and ‘convenience for family’ were the two most frequently cited reasons nulliparous women gave for choosing a unit (59 and 51 % respectively). For multiparous women ‘previous experience’ was the most common reason but ‘distance from home’ and ‘convenience for family’ were jointly the second most commonly cited reasons (44 % in both cases) [21].

### Transfer

Longworth’s study found that women with a ‘dominant preference for hospital birth’ had a significant preference for somewhere without the need for transfer, whereas this was not significant for women with a ‘dominant preference for home birth’ or amongst ‘traders’ [18]; and the findings of Rogers’ study were broadly consistent with this [24]. In Lavender’s survey 28 % of respondents agreed or strongly agreed with the statement “I would not want to transfer to a hospital a few miles away if my baby or I develop a problem” and around half disagreed or strongly disagreed with the statement [22]. However, because of the complex and potentially ambiguous wording of this negative statement it is unclear whether those disagreeing are indicating that they would consider a setting where transfer would be required in the event of complications or merely saying that if complications arose they would want to be transferred to hospital.

### Obstetric unit vs. midwifery unit

Donaldson’s study asked low risk women booked at a hospital with an OU and AMU to choose between two vignettes characterising care in a labour ward vs. care in a midwifery unit (see Table 1): 33 % did not express a preference, 55 % preferred the midwifery unit and 11 % the labour ward [19].

The discrete choice experiment by Pitchforth in remote and rural areas of Scotland found that women preferred consultant-led care over midwife-managed care and were prepared to travel further to access their preferred choice, but that preferences varied by geographical location, with island residents preferring midwifery-led care. Women with ‘high-risk’ episodes during pregnancy were also more likely to prefer consultant-led care. Respondents tended to prefer the model of care that they had experienced during their most recent birth [20].

### Other preferences

Other attributes not noted above that were important to at least 50 % of survey respondents included: having a birth companion present [21, 23], information and being kept informed [21, 23], and having a special care baby unit (SCBU) on site [22].

In one study of AMU users, ‘wants natural childbirth’ and ‘family can be involved’ were reasons mentioned by two thirds of women who had said that they would still choose the unit if it became an FMU [24].

Hundley’s studies explored preferences for intermittent vs. continuous fetal monitoring but findings were contradictory: 78 % of women expressed a preference for intermittent fetal monitoring, but the discrete choice experiment findings suggested that women tended to prefer scenarios with continuous fetal heart rate monitoring [15, 16]. The reasons for this are unclear. Hundley and colleagues suggest that a possible explanation is that respondents may be more likely to give a “socially acceptable” response to a direct question while revealing their true attitudes when trading attributes in the context of a discrete choice experiment, but they also discuss a range of other possible explanations [15].

### Variations in preferences by parity, ethnicity, and level of area deprivation

The included studies provided limited data on whether preferences differed according to the women’s characteristics. In Lavender’s survey women’s views did not differ by age or level of area deprivation. Nulliparous women were more likely to say that the availability of a pool was important to them (32 % vs. 19 %). Compared with white European women, ethnic minority women were significantly more likely to feel unsafe if a doctor was not immediately available (78 % vs. 60 %) and were more likely to consider it important to have a SCBU available where they gave birth (84 % vs. 73 %) [22]. Donaldson’s findings suggested that women across social classes were more likely to prefer a midwifery unit to a labour ward but with a possible trend towards more women in higher social classes having a preference for midwifery unit birth [19]. Several studies found that multiparous women’s choices appeared to be influenced by their previous birth experience [20, 21, 24].

## Discussion

### Main findings

The main findings are summarised in Table 3.

**Table 3:** Summary of main findings

Attribute of care	Women’s birth place preferences
Pain relief	Women attach considerable importance to the availability of pain relief options. Some wish to have access to an epidural if needed, without necessarily intending to have one.
	Pain relief preferences appear to be influenced by women’s expectations of the options available to them.
Medical staff involvement/availability	A substantial proportion of women have a strong preference for care in a hospital setting where medical staff are not necessarily involved in their care, but are readily available.
	Ethnic minority women may be more likely to prefer a hospital birth and to have a range of medical facilities available on site.
‘Homely’ environment/atmosphere	Women tend to prefer more homely environments but preferences may be weaker than for other attributes.
Style of decision-making	Many women attach considerable importance to models of decision-making in which the woman is involved in decisions about her care.
Distance	Proximity of services and/or travel time are important considerations for most women. Many women have a preference for a local unit and in some instances will trade off other preferences in order to attend a local unit, but women who have a strong preference for a consultant-led unit (or for specific services only available in a hospital with an OU) will travel further in order to access a unit where they feel safe.
	Women living in remote areas may accept long travel times whereas women living in urban areas where hospitals are typically closer may be less prepared to travel.
	Nulliparous women may be willing to travel further to a maternity unit that they perceive provides ‘higher quality care’.
Transfer	Women who prefer a hospital birth tend to express concern about transfer, whereas women who prefer a midwifery-led setting tend to be less concerned about transfer.
Other	Having a birth companion present, information and being kept informed, a quiet atmosphere, and having a special care baby unit (SCBU) on site are amongst other attributes found to be important.

### Findings from other countries

The preferences summarised in Table 3 were elicited from women in the UK, but these findings appear to be broadly consistent with findings from similar quantitative studies in other countries with established midwifery-led birth options.

In the Netherlands, three discrete choice experiments - two linked studies [25, 26] and a small pilot study [27] - have been conducted in a cohort of low-risk nulliparous women. These investigated seven attributes, many of which were informed by the attributes considered in the UK studies covered in this review [16, 18]. These attributes included: assistance by a midwife versus an obstetrician during birth, home-like versus clinical ‘ambience’, possibility of influencing decision making during birth, possibility of ‘pain-relief treatment during birth’, place of birth (home vs. hospital), need for transport in case of complications. Consistent with our findings for UK women, influence on decision-making was important to women irrespective of their planned birth setting, as was the availability of pain-relief treatment. Giving birth in hospital was strongly preferred by women planning an obstetric-led hospital birth. Women planning a midwife-led hospital birth (equivalent to an AMU in the UK) also had a significant preference for being in hospital [25]. Two of the studies [26, 27] additionally considered interactions with socioeconomic variables. These analyses showed, for example, that women with higher educational attainment had a stronger preference for involvement in decision making and for the availability of pain-relief treatments than those with lower educational attainment.

A descriptive survey in the same cohort of nulliparous women in the Netherlands [28] explored women’s motives for preferring each of the three settings available. Again, findings were broadly consistent with those summarised in this review. For example, the lack of ready availability of specialist help and concerns about transfer were important reasons for women preferring midwife-led and obstetric-led hospital births.

Findings from other countries are sparser. A study in New Zealand [29] which investigated what influenced women to choose an FMU versus a tertiary hospital unit found that women who chose an FMU were most strongly influenced by proximity and ease of access and by the atmosphere or ‘feel’ of the unit and were largely uninfluenced by the lack of specialist services. In contrast, the women who chose a hospital birth were mainly influenced by the availability of specialist services and by their confidence in hospital staff, and distance/ease of access played a smaller part. Again, these findings confirm that women differ with regards to their preference for availability of medical staff, with some preferring on site medical staff and others unconcerned about this as observed in the UK [22]. A further survey conducted in rural Tasmania where women had to travel between 45 min and 2 h to the nearest major hospital also found that women traded off distance against perceived safety with the majority of women (59 %) prepared to travel less than an hour to access ‘safe delivery’ [30]. These findings are also consistent with Pitchforth’s findings that women in remote and rural areas of the UK are potentially prepared to travel further to access hospital care but that women switch to less preferred options when the distance becomes too great [20].

Finally, a study in Canada [31] that investigated how pregnant women decided between home or hospital birth in Ontario province found that, overall, the top priorities for women when making a choice were feeling safer, feeling more comfortable and seeing birth as a natural process, but priorities differed depending on whether the woman had a preference for a home or hospital birth. The top priorities for women preferring a hospital birth were feeling safer, wanting access to pain medication and feeling more comfortable; while the top priorities of those wanting a home birth were seeing birth as a natural process, wanting to avoid interventions, and feeling more comfortable. The vast majority of respondents in both groups also stated that they wanted to be involved in decision making. The study also included a small ‘undecided’ group. These women all wanted to avoid interventions but other preferences and priorities were mixed and more than half thought that hospital birth was safer for the baby than home birth.

Overall the consistency of findings appears to suggest that the birth place preferences described in this review may well be generalisable to other high-income countries with established midwifery-led birth options similar to those in the UK.

### Strengths and limitations of the review

The main strengths of this review are that we have systematically identified and synthesised the quantitative evidence from reports published in scientific journals since 1992, providing evidence gathered from women in the UK about their birth place preferences.

A limitation is that, for pragmatic reasons, we took the decision to include only reports published in scientific journals and have not included grey literature or doctoral theses. As a check on possible gaps, we reviewed reports of recent national surveys of maternity care that have included questions relating to choice of place of birth [32–36] and other reports known to the authors that have addressed questions relating to choice [7, 37]. Only one of these [36] contained relevant data, although the survey methods (an online survey of members of the NCT, a national charity that provides support to parents) were such that the generalisability of the findings is uncertain. In this survey the vast majority (90 %) of respondents were having a first baby so the findings largely reflect the preferences of this group and results suggest a possible bias towards women preferring non-OU settings. More than half of respondents wanted to give birth in an AMU (broadly consistent with Donaldson’s findings [19]), 10 % at home (slightly higher than the proportions considering home birth reported by Lavender (7.6 %) and Longworth (3 %) [18, 22]), 25 % in an OU and 6 % in an FMU. Interestingly, the overall proportion of respondents wanting an FMU birth was 7 % or less for women having a first, second or third baby. However, in areas where an FMU was a possible choice, 21 % of women reported that they wanted an FMU birth. Facilities (e.g. birth pools and partner accommodation) and safety were the most commonly mentioned reasons for choosing a particular location. Availability of medical staff and technology appeared to be particularly important to women choosing an OU, some of whom reported they didn’t feel comfortable going elsewhere whereas the unit being ‘friendly and supportive’, the availability of specific facilities, ‘less medical intervention’ and knowing that the location gave ‘great care’ were more frequently reported by women wanting an FMU birth. These findings appear to be broadly consistent with those of the studies included in this review.

We also carried out keyword searches of the NICE NHS Evidence database [38] and the British Library EThOS database of doctoral theses [39] but did not identify any further relevant quantitative studies. For these reasons we do not believe that extending our searches and inclusion criteria to other report types would have added important data to this quantitative review although we may have failed to include some relevant studies, particularly local surveys.

A further limitation is the generally poor methodological quality of the included studies. However, although many of the surveys were small and conducted in single units or small geographical areas, the consistency of some descriptive findings across studies suggests a degree of generalisability. Quality appraisal indicated that the experimental stated preference studies were of average quality by current standards partly reflecting the fact that the design, conduct and reporting of stated preference studies have advanced since the time these studies were conducted.

The relevance of the findings of this review to policy and practice is somewhat limited by the age of many of the included studies and the paucity of recent evidence. For example, only one of the included reports [24] is based on data collected since the publication of Maternity Matters in 2007 [2]. A further issue related to the age of the included studies is the extent to which women’s beliefs and preferences may have been influenced by the availability of new evidence indicating the safety and benefits of midwifery-led birth settings for low risk women [40, 41] and by the recent expansion in the provison of midwifery units, particularly AMUs [7]. The extent to which women’s beliefs and preferences may be influenced by evidence and possibly by the increasing normalisation of midwifery-led settings is not well understood, but initiatives such as the Birth Place Choices Project [42, 43] have demonstrated that the acceptability and uptake of midwifery-led options is not fixed and can be influenced by measures such as providing training and support for midwives to ensure that the information and guidance given to women about the available choices is evidence-based. It is therefore possible that the data presented here may no longer fully reflect women’s current preferences. Indeed, much of the expansion in midwifery-led care has taken place in the period for which we have little evidence: in 2007 just over 3 % of trusts had an OU, AMU and FMU [44], whereas BirthChoiceUK data for 2015 indicate that currently 17 % of trusts in England provide all three options (Miranda Dodwell, personal communication).

A further limitation of the literature is that it provides very little evidence that directly illuminates women’s preferences for different types of maternity unit, e.g. AMU vs. FMU. Attributes such as availability of medical staff, availability of epidural pain relief and preferences for a setting where transfer will not be required if complications develop capture some but not necessarily all of the attributes that women may consider important when making a choice between an AMU and an FMU.

Six of the nine included studies were conducted in Scotland with several conducted in the same region in and around a relatively small city in eastern Scotland; and notably four of the five stated preference studies were conducted in Scotland. Findings, particularly those relating to distance and transfer, may therefore have been influenced by the fact that the Scottish study participants were drawn from less urbanised and more sparsely populated areas of the UK; and preferences relating to units with and without onsite medical staff may also have been influenced by the fact that FMUs (known as ‘community maternity units’ in Scotland) have been an established feature of Scottish maternity care for many years.

The review has identified some gaps in the quantitative evidence required by policy makers and service planners. For example, our findings suggest that there may be a mismatch between existing patterns of service provision and women’s preferences, with potentially more women having preferences that might be better met by birth in a midwifery unit than an OU, but we cannot reliably estimate from available data what proportion of low risk women have a preference for birth in a midwifery unit or conversely what proportion of low risk women currently have a preference for birth in an obstetric unit with the associated reassurance of immediate access to medical staff.

Findings from the UK and elsewhere also suggest that preferences for some attributes have not been adequately explored. For example, with the exception of the study by Donaldson which did not investigate individual attributes, none of the stated preference studies explored attributes such as unit intervention rates or other attributes that women seeking a ‘non-medicalised birth’ might prefer. Further research exploring women’s preferences for settings achieving low intervention rates might be merited.

## Conclusions

The findings of this review suggest that there are some service attributes that are valued by the vast majority of women. These include local services, being attended by a known midwife throughout labour and, for most but not all women, a preference for a degree of control and involvement in decision-making. Women’s views and preferences differ markedly for other attributes, such as availability and degree of involvement of medical staff, the availability of epidural vs. other pain relief options and a ‘homely’ vs. clinical appearance of the delivery rooms. This suggests that policy makers, commissioners and service providers might usefully consider how to extend the availability of services that most women value while offering a choice of options that enable women to access services that best fit their needs and preferences. However, there is good evidence that preferences are influenced by expectations so it is important to recognise that women’s preferences and the birthplace decisions that they take may be influenced by the fact that OU births are currently the norm.

## Abbreviations

AMU, alongside midwifery unit; FMU, freestanding midwifery unit; ISPOR, International Society for Pharmacoeconomics and Outcomes Research; OU, obstetric unit; SCBU, special care baby unit

## Additional files

Additional file 1: Search and study selection strategy. Details of: search strategy, screening and study selection procedures. (DOCX 24 kb) Additional file 2: Description of included studies. Description of included quantitative studies – expanded version of Table 1. (DOCX 47 kb) Additional file 3: Critical appraisal of included surveys. Includes critical appraisal findings for the seven included surveys and an overview of the strengths an limitations of the included surveys. (DOCX 23 kb) Additional file 4: Quality appraisal of stated preference studies. Describes the findings of the critical appraisal of the five included stated preference studies using an ISPOR checklist. (DOCX 71 kb) Additional file 5: Narrative summary of findings from each of the included studies. The file provides a table summarising results from each study relating to (a) stated preferences and (b) factors that women report influenced their choice of unit or birth setting. (DOCX 36 kb)

## References

[1] National Institute for Health and Clinical Excellence (2014). Clinical guideline 190: intrapartum care for healthy women and babies.

[2] Department of Health (2007). Maternity matters: choice, access and continuity of care in a safe service.

[3] Department of Health (1993). Changing childbirth, part 1: report of the expert maternity group.

[4] Department of Health Social Services and Public Safety (DHSSPS): A Strategy for Maternity Care in Northern Ireland 2012-2018. In: A Strategy for Maternity Care in Northern Ireland 2012-2018. Belfast: DHSSPS; 2012. https://www.health-ni.gov.uk/publications/strategy-maternity-care-northern-ireland-2012-2018. Accessed 5 May 2016.

[5] The Maternity Services Action Group: A Refreshed Framework for Maternity Care in Scotland. In: A Refreshed Framework for Maternity Care in Scotland. Edinburgh: The Scottish Government; 2011. http://www.gov.scot/Publications/2011/02/11122123/11. Accessed 5 May 2016.

[6] Welsh Government: A strategic vision for maternity services in Wales. In: A strategic vision for maternity services in Wales. WG12896. Welsh Govenment. 2011. http://gov.wales/topics/health/publications/health/strategies/maternity/. Accessed 5 May 2016.

[7] National Audit Office (2013). Maternity services in England. Report by the comptroller and auditor general.

[8] Care Quality Commission: 2015 survey of women’s experiences of maternity care. Statistical release. In: 2015 survey of women’s experiences of maternity care. Care Quality Commission. 2015. http://www.cqc.org.uk/content/maternity-services-survey-2015. Accessed 5 May 2016.

[9] Office for National Statistics: Birth Characteristics in England and Wales, 2014. In: ONS Statistical Bulletin Birth Characteristics in England and Wales. Office for National Statistics; 2015. http://www.ons.gov.uk/ons/dcp171778_384394.pdf. Accessed 25 Nov 2015.

[10] BirthChoiceUK for the Royal College of midwives: Trends in Freestanding Midwife-led Units in England and Wales 2001-2013. In: Trends in Freestanding Midwife-led Units in England and Wales 2001-2013. Royal College of Midwives; 2013. https://www.rcm.org.uk/sites/default/files/FMU%20Trends%20-%20Web%20Final.pdf. Accessed 23 Nov 2015

[11] Cooke A, Smith D, Booth A (2012). Beyond PICO: the SPIDER tool for qualitative evidence synthesis. Qual Health Res.

[12] Centre for Evidence-Based Management (CEBMa): Critical Appraisal of a Survey. Critical appraisal tool : http://www.cebma.org/wp-content/uploads/Critical-Appraisal-Questions-for-a-Survey.pdf Accessed 9 Sept 2015.

[13] Bridges JF, Hauber AB, Marshall D, Lloyd A, Prosser LA, Regier DA, Johnson FR, Mauskopf J (2011). Conjoint analysis applications in health—a checklist: a report of the ISPOR good research practices for conjoint analysis task force. Value Health.

[14] Thomas J, Brunton J, Graziosi S (2010). EPPI-reviewer 4.0: software for research synthesis.

[15] Hundley V, Ryan M (2004). Are women’s expectations and preferences for intrapartum care affected by the model of care on offer?. BJOG.

[16] Hundley V, Ryan M, Graham W (2001). Assessing women’s preferences for intrapartum care. Birth.

[17] Ratcliffe J, Longworth L (2002). Investigating the structural reliability of a discrete choice experiment within health technology assessment. Int J Technol Assess Health Care.

[18] Longworth L, Ratcliffe J, Boulton M (2001). Investigating women’s preferences for intrapartum care: home versus hospital births. Health Soc. Care Community.

[19] Donaldson C, Hundley V, Mapp T (1998). Willingness to pay: a method for measuring preferences for maternity care?. Birth.

[20] Pitchforth E, Watson V, Tucker J, Ryan M, van Teijlingen E, Farmer J, Ireland J, Thomson E, Kiger A, Bryers H (2008). Models of intrapartum care and women’s trade-offs in remote and rural Scotland: a mixed-methods study. BJOG.

[21] Emslie MJ, Campbell MK, Walker KA, Robertson S, Campbell A (1999). Developing consumer-led maternity services: a survey of women’s views in a local healthcare setting. Health Expect.

[22] Lavender T, Chapple J (2005). How women choose where to give birth. Pract. Midwife.

[23] Rennie A-M, Hundley V, Gurney E, Graham W (1998). Women’s priorities for care before and after delivery. British J Midwifery.

[24] Rogers C, Harman J, Selo-Ojeme D (2011). Perceptions of birth in a stand-alone centre compared to other options. British J Midwifery.

[25] van Haaren-ten Haken T, Pavlova M, Hendrix M, Nieuwenhuijze M, de Vries R, Nijhuis J (2014). Eliciting preferences for key attributes of intrapartum care in the Netherlands. Birth.

[26] Hendrix M, Pavlova M, Nieuwenhuijze MJ, Severens JL, Nijhuis JG (2010). Differences in preferences for obstetric care between nulliparae and their partners in the Netherlands: a discrete-choice experiment. J. Psychosom. Obstet. Gynaecol.

[27] Pavlova M, Hendrix M, Nouwens E, Nijhuis J, van Merode G (2009). The choice of obstetric care by low-risk pregnant women in the Netherlands: Implications for policy and management. Health Policy.

[28] van Haaren-ten Haken T, Hendrix M, Nieuwenhuijze M, Budé L, de Vries R, Nijhuis J (2012). Preferred place of birth: characteristics and motives of low-risk nulliparous women in the Netherlands. Midwifery.

[29] Grigg C, Tracy SK, Daellenbach R, Kensington M, Schmied V (2014). An exploration of influences on women’s birthplace decision-making in New Zealand: a mixed methods prospective cohort within the evaluating maternity units study. BMC Pregnancy Childbirth.

[30] Hoang H, Le Q (2012). Trade-off between local access and safety considerations in childbirth: rural Tasmanian women’s perspectives. Aust. J. Rural Health.

[31] Murray-Davis B, McDonald H, Rietsma A, Coubrough M, Hutton E (2014). Deciding on home or hospital birth: results of the Ontario choice of birthplace survey. Midwifery.

[32] Healthcare Commission (2008). Towards better births a review of maternity services in England.

[33] Redshaw M, Heikkila K (2010). Delivered with care: a national survey of women’s experience of maternity care 2010.

[34] Redshaw M, Henderson J. Safely delivered: a national survey of women’s experience of maternity care 2014. Oxford: National Perinatal Epidemiology Unit; 2015.

[35] Redshaw M, Rowe R, Hockley C, Brocklehurst P (2010). Recorded delivery: a national survey of women’s experience of maternity care 2006.

[36] Bourke G (2013). Support overdue: women’s experiences of maternity services.

[37] Dodwell M, Gibson R (2009). An investigation into choice of place of birth.

[38] Evidence search, Health and social care (online database) [www.evidence.nhs.uk] Accessed 23 Nov 2015

[39] EThOS: UK E-Theses Online Service (online database) [http://www.bl.uk/reshelp/findhelprestype/theses/ethos/] Accessed 23 Nov 2105

[40] Brocklehurst P, Hardy P, Hollowell J, Linsell L, Macfarlane A, McCourt C, Marlow N, Miller A, Newburn M, Birthplace in England Collaborative Group (2011). Perinatal and maternal outcomes by planned place of birth for healthy women with low risk pregnancies: the Birthplace in England national prospective cohort study. BMJ.

[41] Hollowell J, Puddicombe D, Rowe R, Linsell L, Hardy P, Stewart M, Newburn M, McCourt C, Sandall J, Macfarlane A (2011). The birthplace national prospective cohort study: perinatal and maternal outcomes by planned place of birth. Birthplace in England research programme. Final report part 4.

[42] Barber T, Rogers J, Marsh S (2006). The birth place choice project: phase one. British J Midwifery.

[43] Barber T, Rogers J, Marsh S (2007). Increasing out-of-hospital births: what needs to change?. British J Midwifery.

[44] Redshaw M, Rowe R, Schroeder L, Puddicombe D, Macfarlane A, Newburn M, McCourt C (2011). Mapping maternity care. The configuration of maternity care in England. Birthplace in England research programme. Final report part 3.

