# Supplementary material for: Women’s birth place preferences in the United Kingdom: a systematic review and narrative synthesis of the quantitative literature
Source: BMC Pregnancy Childbirth. 2016 Aug 8;16:213. doi: 10.1186/s12884-016-0998-5 (PMC4977690; doi:10.1186/s12884-016-0998-5)
Supplement: Additional file 1: — Search and study selection strategy. Details of: search strategy, screening and study selection procedures. (DOCX 24 kb) [file 12884_2016_998_MOESM1_ESM.docx]

**Additional file 1: Search and study selection strategy**

The quantitative systematic review of birth place preferences was one component of a broader systematic review which also encompasses the qualitative evidence relating to factors that may affect women’s choice of place of birth, including beliefs, preferences, knowledge and experience. Some aspects of the methods reflect the fact that searches were common to both the quantitative and the qualitative reviews.

The search strategy was designed to identify research studies within the scope of the broader review irrespective study design and screening was conducted by sequentially applying the criteria applicable to each component of the broader review as described below. Researchers who were part of the broader research team Kirstie Coxon (KC), the lead author of the qualitative review, Alison Chisholm (AC), a qualitative researcher, and Joanne Forsey (JF), a midwife and MRes student at City University were involved in screening in addition to the authors of the present study.

**1. Search strategy**

We used the a search strategy based on the SPIDER tool [[1](#_ENREF_1)]

| **SPIDER Tool** | **Search Terms relating to:** |
| --- | --- |
| **S**ample | **Pregnant women**  Matern* or pregnan* OR women Pregnant women/ Mothers/ |
| **P**henomenon of **i**nterest | **Maternity unit/midwifery unit/birth centre/home birth/intrapartum care/place of birth**  Maternity adj2 (care or unit* or setting? Or center? Or centre? Or hospital? Or service*) obstetric adj2 (unit? or center? Or centre?) midwi* adj2 (unit? or center? Or centre?) Home birth* or home childbirth or home delivery birth adj2 (unit? or center? Or centre? Or place) intrapartum care  Place of birth/ Birthing Centers/ Delivery Rooms/ Home Childbirth/ |
| **D**esign | NA |
| **E**valuation | **Preferences/choice/experiences/decisions/views/influences/experiences/ attitudes/expectations**  Prefer* or choice* or choos* or option? Or decision* or decid* or view* or experience* or need* or suggest* or influenc* or attitude* or satisf* or value* or expectation* or inform* or advice*or consum* or “Consumer –led” |
| **R**esearch setting | **United Kingdom/Great Britain/England/Scotland/Northern Ireland/United Kingdom/British/NHS**  United kingdom or uk or britain or gb or england or wales or scotland or northern ireland or british or nhs or national health service or Great Britain |

***Databases searched***

- Applied Social Science Index and Abstracts (ASSIA)[Proquest]
- Cumulative Index to Nursing and Allied Health (CINAHL) plus [EBSCOHost]
- EMBASE [OvidSP]
- Medline [OvidSP]
- PsycINFO [OvidSP]
- Science Citation Index [Web of Science Core Collection]
- Social Sciences Citation Index [Web of Science Core Collection]

Databases were searched in mid-March 2015.

**2. Screening and study selection**

Two reviewers independently screened titles and abstracts and full text as required. As noted above, because this review was conducted as one component of a broader systematic review the screening was conducted by sequentially applying the criteria applicable to each component of the review, with reviewers working in pairs. Screening was conducted as follows:

**Stage 1:** Jennifer Hollowell (JH) and JF screened all titles and abstracts applying only the exclusion criteria relating to country, broad topic of research, type of report and study population. Only references independently excluded by both reviewers were excluded. Remaining references were rescreened in stage 2.

**Stage 2:** Remaining tiles/abstracts (n= 487) were re-screened by KC and JH who applied the screening criteria for the broader review, but did not exclude studies solely on the basis of design (i.e. qualitative, mixed-methods and quantitative studies were included). Discrepancies were resolved by discussion. Full-text articles (n=72) were retrieved for the remaining studies.

**Stage 3 (qualitative review):** JH and KC read and independently screened the full-text articles to identify eligible qualitative and mixed-methods studies. Discrepancies were resolved by discussion, with a third reviewer (AC) involved as required. During this screening process, JH and KC flagged all potentially eligible quantitative and mixed-methods studies.

**Stage 4 (quantitative review):** JH and Reem Malouf (RM) independently screened the full-text articles flagged at stage 3 to identify eligible quantitative studies. Discrepancies were resolved by discussion, with a third reviewer Yangmei Li (YL) involved as required.

**References**

1. Cooke A, Smith D, Booth A: **Beyond PICO: the SPIDER tool for qualitative evidence synthesis**. *Qualitative health research* 2012, **22**(10):1435-1443.
