# Supplementary material for: Women’s birth place preferences in the United Kingdom: a systematic review and narrative synthesis of the quantitative literature
Source: BMC Pregnancy Childbirth. 2016 Aug 8;16:213. doi: 10.1186/s12884-016-0998-5 (PMC4977690; doi:10.1186/s12884-016-0998-5)
Supplement: Additional file 2: — Description of included studies. Description of included quantitative studies – expanded version of Table 1. (DOCX 47 kb) [file 12884_2016_998_MOESM2_ESM.docx]

**Additional file 2: Additional details of included study methods**

| **Study ID** | **Study methods** |
| --- | --- |
| **Donaldson (1998)** | Willingness to pay study designed to evaluate 'low risk' women's preference for type of intrapartum care (OU vs. AMU) at around the time of the booking visit. Respondents were provided with a description of the key features of the two types of unit (including style of decision-making, one-to-one care from a midwife, electronic vs. 'traditional' fetal monitoring) and quantitative information about, for example, the chances of transfer, the proportions of women able to move around freely, having an epidural, transferred for an epidural. ‘Low risk’ women were mailed the questionnaire before booking with two subsequent reminders sent three and six weeks later, possibly after the booking visit. |
| **Emslie (1999)** | For the survey component of the study, questionnaires were mailed to women in the FMU's catchment area at around 14 weeks gestation with one reminder letter sent after three weeks. Further questionnaires were sent at 36 weeks gestation and 6 weeks postnatally. This survey was one component of a mixed methods study. |
| **Hundley (2001)** | A discrete choice experiment in which ‘low risk’ pregnant participants were asked to choose between pairs of hypothetical scenarios. These scenarios were based on attributes identified in the literature as potentially important to women and which could vary between units. The attributes covered: continuity of carer (four levels varying in the extent to which the woman would know the midwife providing labour care); pain relief (three levels: all methods, all methods but transfer required for epidural, all methods other than epidural); type of fetal heart rate monitoring (continuous vs. intermittent), appearance of room (homely vs. clinical), involvement of medical staff (yes, vs. only if required), and involvement in decision-making (four levels ranging from no involvement to women deciding). ‘Low risk’ women were recruited at booking in three areas in Grampian (Scotland). Data were collected by postal questionnaire. A reminder system was not possible for data protection reasons. |
| **Hundley (2004)** | A discrete choice experiment in which ‘low risk’ pregnant participants were asked to choose between pairs of hypothetical scenarios. These scenarios were based on attributes identified in the literature as potentially important to women and which could vary between units. See Hundley (2001) for details. Three groups of women were recruited at booking: (a) women booking at Aberdeen Maternity Hospital who were eligible for AMU care; (b) women booked at the FMU in Peterhead Community Hospital; and (c) ‘comparable’ women booked at Dr Gray's Hospital, Elgin, a hospital providing ‘shared care’ (obstetricians and midwives at the hospital and GP/midwives in the community) with medical interventions available but without an epidural service. Data were collected by postal questionnaire. A reminder system was not possible for data protection reasons |
| **Lavender (2005)** | A survey of pregnant women in a purposive sample of 12 maternity units in England. Units were sampled to ensure the inclusion of units serving women from various socio-economic/ethnic backgrounds and from urban and rural areas. Units were included that offered different birth settings (home, FMU, AMU and OU) and varied in size (50 births to 6000 births). The study sample appears to have been a cross-sectional sample of women receiving antenatal care. The survey questionnaire included both open and closed questions and a series of statements that women were asked to either agree or disagree with. This survey was one component of a mixed methods study. |
| **Longworth (2001)** | The study used conjoint analysis to assess preferences for different aspects of intrapartum care comparing women actively choosing home birth to women who had booked for a hospital delivery. Literature and focus groups were used to identify attributes that were important to women. Scenarios based on these attributes were developed covering: continuity of contact with the same midwifery staff (unknown midwife, have previously met midwives, midwife well known to woman), location (labour ward, midwifery unit, home), pain relief (gas and air only, gas and air+ birthing pool, all types of pain relief including epidural), decision-making (by medical staff, shared, by woman), transfer (none, low probability, high probability). Two samples of women were selected from each of the two maternity units: (a) women who had booked for a home delivery; (b) 'low risk' women who had booked for a hospital delivery. The women appear to have been surveyed by postal questionnaire postnatally with one reminder sent to non-respondents after 4 weeks. |
| **Pitchforth (2008)** | The study involved a discrete choice experiment in which women were asked to choose between hypothetical scenarios. Three attributes were varied in eight different scenarios: type of unit (midwife-led (MU) vs. consultant-led (OU)), pain relief (all methods vs. no epidural) and travel time to unit (home (0 mins) vs. 30 mins from home vs. 60 mins vs. 90 mins vs. 120 mins). Eight small maternity units (<300 births per annum) were purposively selected to provide a spread of staffing/service models. The sample included four community FMUs and one FMU adjacent to a non-obstetric hospital, one GP-run community maternity unit, and two consultant-led units (OUs) both without neonatal facilities. Women resident in the catchment areas of these units who gave birth in the study period were sent a postal questionnaire six weeks after the birth with one reminder two weeks later. Women who had delivered in three non-study obstetric units in the region were included. These non-study OUs included two with neonatal facilities and one without. Data were also collected from medical notes on participants' risk status during pregnancy and at the time of the birth. |
| **Rennie (1998)** | A questionnaire survey in which a stratified sample of women expecting a first or second baby, living within Aberdeen city and booked for delivery in Aberdeen Maternity Hospital (OU or AMU) were recruited in antenatal care at 34 weeks. A follow-up questionnaire was given to women to complete at home 10 days after the birth. Follow-up of non-responders included an initial telephone call and a second postal reminder if required and was the same antenatally and postnatally. Additional data were extracted from medical notes. |
| **Rogers (2011)** | A questionnaire survey was conducted amongst a cross-sectional sample of 'AMU users': women who were either booked, considering booking or who had given birth at the AMU situated in a hospital where a relocation of the OU was planned. |

.
