# Supplementary material for: Women’s birth place preferences in the United Kingdom: a systematic review and narrative synthesis of the quantitative literature
Source: BMC Pregnancy Childbirth. 2016 Aug 8;16:213. doi: 10.1186/s12884-016-0998-5 (PMC4977690; doi:10.1186/s12884-016-0998-5)
Supplement: Additional file 3: — Critical appraisal of included surveys. Includes critical appraisal findings for the seven included surveys and an overview of the strengths an limitations of the included surveys. (DOCX 23 kb) [file 12884_2016_998_MOESM3_ESM.docx]

**Additional file 3. Critical appraisal of included studies**

Based on a modified version of the Centre for Evidence Based Management ‘*Critical Appraisal of a survey’* tool^[[1]](#endnote-1)^. Modified wording of appraisal questions is shown in italics.

**Table 1. Critical appraisal of included surveys**

| **Appraisal question**  Key: | | | | Donaldson (1998) | Emslie (1999) | Lavender (2005) | Hundley (2001) | Hundley (2004) | Rennie (1998) | Rogers (2011) |
| --- | --- | --- | --- | --- | --- | --- | --- | --- | --- | --- |
| Y | Yes | (Y) | Yes with limitations |  |  |  |  |  |  |  |
| N | No (poor) | ? | Not reported or unclear |  |  |  |  |  |  |  |
| 1. Did the study address a clearly focused question *relating to birth place preferences?* | | | | Y | Y | Y | Y | Y | (Y) | Y |
| 2. Is the research method (study design) appropriate for answering the research question? | | | | Y | Y | Y | Y | Y | Y | Y |
| 3. Is the method of selection of the participants (employees, teams, divisions, organizations) clearly described? | | | | Y | Y | (Y) | Y | Y | Y | Y |
| 4. Could the way the sample was obtained *be free from* (selection) bias? | | | | (Y) | (Y) | (Y) | (Y) | (Y) | N | N |
| 5. Was the sample of participants representative with regard to the population to which the findings will be referred? | | | | (Y) | (Y) | (Y) | (Y) | (Y) | N | (Y) |
| 6. Was the sample size based on pre-study considerations of statistical power? | | | | ? | ? | ? | Y | Y | ? | ? |
| 7. Was a satisfactory response rate achieved? | | | | Y | Y | Y | N | N | Y | N |
| 8. Are the measurements (questionnaires) likely to be valid and reliable? | | | | ? | ? | Y | Y | Y | Y | (Y) |
| 9. Was the statistical significance assessed? | | | | (Y) | (Y) | (Y) | N | Y | Y | Y |
| 10. Are confidence intervals given for the main *descriptive survey* result*s*? | | | | N | N | N | N | N | N | (Y) |
| 11. *Was the sample size adequate?* | | | | N | N | Y | (Y) | (Y) | N | N |
| 12. Can the results be *generalized to UK low risk women in general or to other specific groups of women?* | | | | N | N | (Y) | N | N | N | N |

Studies conducted by Longworth et al. (2001) and Pitchforth et al. (2008) were not included in this appraisal because no descriptive survey data on preferences were reported. See supplementary file 4 for critical appraisal of stated preference studies.

**Table 2. Summary description of strengths and limitation of the included surveys**

| *Donaldson* | Single centre, relatively small sample size, good response rate. No confidence intervals reported for descriptive statistics. |
| --- | --- |
| *Emslie* | Single area, relatively small sample size, good response rate in early pregnancy survey, lower response rate in follow-up survey at 36 weeks. |
| *Lavender* | Survey based on nationally representative sample of units with apparently adequate response rate, but uncertainty about possible selection bias. Limited statistical analysis and no confidence intervals presented for descriptive statistics. |
| *Hundley 2001* | Single region with three centres. Poor response rate, relatively small sample, no confidence intervals. |
| *Hundley 2004* | See Hundley 2001. |
| *Rennie* | Relatively small, single centre study with good response rate. Questionnaire not well described making some results difficult to interpret. |
| *Rogers* | Small single centre study with relatively low response rate. |

1. http://www.cebma.org/wp-content/uploads/Critical-Appraisal-Questions-for-a-Survey.pdf [↑](#endnote-ref-1)
