# Supplementary material for: Women’s birth place preferences in the United Kingdom: a systematic review and narrative synthesis of the quantitative literature
Source: BMC Pregnancy Childbirth. 2016 Aug 8;16:213. doi: 10.1186/s12884-016-0998-5 (PMC4977690; doi:10.1186/s12884-016-0998-5)
Supplement: Additional file 4: — Quality appraisal of stated preference studies. Describes the findings of the critical appraisal of the five included stated preference studies using an ISPOR checklist. (DOCX 71 kb) [file 12884_2016_998_MOESM4_ESM.docx]

**Additional file 4: Quality appraisal of stated preference studies**

**Methods**

Five papers reporting stated preference studies were identified by the systematic review – four discrete choice experiments (DCEs) and one willingness-to-pay (WTP) study [[1-5](#_ENREF_1)]. The quality of the methods used in each of these studies was evaluated using the guidelines for conjoint analysis developed by the International Society for Pharmacoeconomics and Outcomes Research (ISPOR) Preference-based Methods Special Interest Group’s Conjoint Analysis Working Group [[6](#_ENREF_6)]. The authors of these guidelines note that although the term ‘conjoint analysis’ is used, the guidelines are equally applicable to DCE studies.

These guidelines present a checklist which contains 30 items, spread over 10 broad research questions (**Table 1**). For each item on the checklist, studies were assigned a score of one if it was fully addressed, 0.5 if it was partially addressed, zero if it was not addressed, or no score if the item was not applicable (N/A). All items were weighted equally and studies were classified as low quality (fulfilled <50% of applicable quality criteria), average quality (fulfilled between 50% and 80% of applicable criteria), or high quality (fulfilled >80% of applicable criteria) [[7](#_ENREF_7)].

Each of the papers evaluated in the quality appraisal presented specific challenges. These are discussed below.

Hundley *et al.* (2001) and Hundley and Ryan (2004) [[2](#_ENREF_2), [3](#_ENREF_3)]

These papers use the same dataset to address two different research questions. As a result, both papers were scored separately in this review. However, credit was given to the later paper if readers were referred to the earlier paper for specific pieces of information that addressed the quality criteria.

Donaldson *et al.* (1998) [[1](#_ENREF_1)]

This paper does not report the results of a DCE or conjoint analysis, rather respondents are asked just one WTP question. As a quality checklist specifically for WTP studies was not identified in the literature, the Bridges *et al.* checklist was adapted in order to evaluate this paper. Only those quality criteria which were not DCE specific were considered (18 of the original 30 criteria), with the quality score expressed as a percentage of these criteria only. Some criteria were DCE specific but could be adapted to consider WTP studies instead (e.g. item 1.3). This paper was therefore assessed against an adapted version of these items.

Longworth *et al.* (2001) [[4](#_ENREF_4)]

This paper reports the main results of a DCE study. It was supplemented in 2002 by a paper by Ratcliffe and Longworth which considered a specific issue concerning DCE experiment design [[8](#_ENREF_8)]. This second paper was not included in the quality appraisal due to its methodological focus, but it was reviewed in order to assess whether any additional information was provided which might change the quality score given to the Longworth *et al.* (2001) paper. However, no additional information was identified.

Pitchforth *et al.* (2008) [[5](#_ENREF_5)]

This paper reports the main results of a DCE study. It was supplemented in 2009 by a paper, again by Pitchforth and colleagues, which reported the results of qualitative work undertaken alongside the DCE [[9](#_ENREF_9)]. This second paper was not included in the quality appraisal as it did not report stated preferences, but it was reviewed in order to assess whether any additional information was provided which might change the quality score given to the Pitchforth *et al.* (2008) paper. However, no additional information was identified.

Analysis

The scores for each paper were calculated individually and then combined in order to calculate an unweighted average quality score across all five studies. As this average score will be biased by the inclusion of two studies using the same dataset [[2](#_ENREF_2), [3](#_ENREF_3)], an adjusted average was also calculated by combining the quality scores for these two papers (taking the highest score for each item across the two papers) and then calculating an overall score. The percentage of studies meeting each of the thirty criteria in the Bridges *et al.* checklist was also calculated. Finally, the results were analysed at the broader research question level in order to identify any general patterns across all the studies.

**Results**

**Tables 2 and 3** present the quality scores for all studies, broken down by item and by research question. The **appendix** contains the detailed checklists completed for each paper. The mean quality score was 55% (range: 44-61%), indicating an evidence base of average quality. When the two Hundley studies were considered as one study, the score for this combined study was 68%, which raised the average quality score across all papers to 56%.

A number of quality criteria were met by most if not all studies. No studies included an opt-out (item 3.3) but this decision was justified in all cases. Almost all studies appropriately justified the sampling strategy that was used (item 7.1), and study limitations, generalisability and implications were generally adequately discussed (items 9.3 and 10.3). However, several quality criteria were met by very few studies. No studies justified the number of attributes or profiles in each choice task (items 3.1 and 3.2), only one study partially described the study data collection instrument and methods (item 10.2) and only one study partially evaluated the properties of the experimental design (item 4.2). Other general weaknesses included a lack of justification for attribute selection (item 2.2), experimental design (item 4.1) or mode of administration (item 7.2), and little consideration of the quality of responses (item 8.2). Specific comments regarding the quality of each paper are provided in the following section.

Hundley *et al.* (2001) and Hundley and Ryan (2004) [[2](#_ENREF_2), [3](#_ENREF_3)]

Hundley *et al.* (2001) is a paper of average quality, with a score of 61%. This paper scored highly on criteria related to the data collection plan: the sampling strategy was broadly justified, all key limitations were noted, and the mode of administration was probably appropriate. Other strengths included the use of additional qualifying questions, the overall level of burden of the data-collection instrument and the inclusion of a comprehensive discussion section which covered study limitations, generalisability and implications. This paper scored poorly on criteria related to the construction of choice tasks, with no justification provided for the number of attributes and profiles selected. Other limitations included a poorly described data-collection instrument and a limited discussion of issues surrounding the experimental design.

Hundley and Ryan (2004) is also a paper of average quality, with a score of 61%, and shares similar strengths and weaknesses with Hundley *et al.* (2001). The key differences are that in this later study the research question and study importance are more precisely defined, but less information is provided on the experimental design and the quality of the responses received. The combined quality score across both Hundley papers is 68%, which indicates that taken as a whole, this study is of average quality.

Donaldson *et al.* (1998) [[1](#_ENREF_1)]

This is a low quality paper, with a score of 44%, although it should be noted that this was a WTP study, so only 18 of the 30 DCE quality criteria were relevant. Only three criteria were fully met: the study rationale was described in detail, the WTP questions were explained well, and the study limitations and generalisability were adequately discussed. Numerous criteria were only partially met (or not met at all), and the data collection plan was particularly poorly described (items 7.1, 7.2 and 7.3).

Longworth *et al.* (2001) [[4](#_ENREF_4)]

This is a paper of average quality, with a score of 57%. This paper scored highly on criteria related to the definition of the research question and the level of justification provided for the use of a DCE. Other strengths included the justification of the sampling strategy and the use of additional qualifying questions to place the DCE results in context. This paper scored poorly on criteria related to the study presentation, the construction of the choice tasks and the experimental design process. In addition, little information was provided on study limitations and generalisability.

Pitchforth *et al.* (2008) [[5](#_ENREF_5)]

This is a low quality paper, with a score of 48%. This paper scored highly on criteria related to the validity of the results and conclusions and the overall presentation of the study. Respondent characteristics were also considered in detail in the analysis. However, this paper scored poorly on criteria related to the selection of attributes and levels, the construction of the choice tasks and appropriate elicitation of preferences.

**Summary**

These results indicate that the stated preference evidence base in this context is of average quality. However, a number of caveats should be noted. First, a checklist published in 2011 was used to evaluate the quality of each paper. This checklist reflects the latest consensus regarding good practice in this field but all of the papers included in this quality appraisal were published before the checklist was devised, hence it is not unreasonable that these papers do not meet all of the quality criteria. Second, when papers have not met one of the items on the checklist this may not necessarily indicate poor quality, rather it may reflect practical issues. For example, it is now common practice for papers reporting the results of DCE studies to publish data collection instruments as electronic supplementary materials. However, this was not necessarily possible for older DCE studies. Finally, some of the DCE studies reported relatively simple experimental designs and did not estimate complex regression models. However, methods for selecting experimental designs have evolved considerably in the past decade, and new approaches for estimating choice models have also been developed (e.g. latent class analysis). Again, it is not necessarily unreasonable that these papers have not met these quality criteria when judged by the very latest standards.

**Table 1: Checklist items**

| **Research question** | **Item** |
| --- | --- |
| 1. Was a well-defined research question stated and is conjoint analysis an appropriate method for answering it? | 1.1 Were a well-defined research question and a testable hypothesis articulated? |
|  | 1.2 Was the study perspective described, and was the study placed in a particular decision-making or policy context? |
|  | 1.3 What is the rationale for using conjoint analysis to answer the research question? |
| 2. Was the choice of attributes and levels supported by evidence? | 2.1 Was attribute identification supported by evidence (literature reviews, focus groups, or other scientific methods)? |
|  | 2.2 Was attribute selection justified and consistent with theory? |
|  | 2.3 Was level selection for each attribute justified by the evidence and consistent with the study perspective and hypothesis? |
| 3. Was the construction of tasks appropriate? | 3.1 Was the number of attributes in each conjoint task justified (that is, full or partial profile)? |
|  | 3.2 Was the number of profiles in each conjoint task justified? |
|  | 3.3 Was (should) an opt-out or a status-quo alternative (be) included? |
| 4. Was the choice of experimental design justified and evaluated? | 4.1 Was the choice of experimental design justified? Were alternative experimental designs considered? |
|  | 4.2 Were the properties of the experimental design evaluated? |
|  | 4.3 Was the number of conjoint tasks included in the data-collection instrument appropriate? |
| 5. Were preferences elicited appropriately, given the research question? | 5.1 Was there sufficient motivation and explanation of conjoint tasks? |
|  | 5.2 Was an appropriate elicitation format (that is, rating, ranking, or choice) used? Did (should) the elicitation format allow for indifference? |
|  | 5.3 In addition to preference elicitation, did the conjoint tasks include other qualifying questions (for example, strength of preference, confidence in response, and other methods)? |
| 6. Was the data collection instrument designed appropriately? | 6.1 Was appropriate respondent information collected (such as sociodemographic, attitudinal, health history or status, and treatment experience)? |
|  | 6.2 Were the attributes and levels defined, and was any contextual information provided? |
|  | 6.3 Was the level of burden of the data-collection instrument appropriate? Were respondents encouraged and motivated? |
| 7. Was the data-collection plan appropriate? | 7.1 Was the sampling strategy justified (for example, sample size, stratification, and recruitment)? |
|  | 7.2 Was the mode of administration justified and appropriate (for example, face-to-face, pen-and-paper, web-based)? |
|  | 7.3 Were ethical considerations addressed (for example, recruitment, information and/or consent, compensation)? |
| 8. Were statistical analyses and model estimations appropriate? | 8.1 Were respondent characteristics examined and tested? |
|  | 8.2 Was the quality of the responses examined (for example, rationality, validity, reliability)? |
|  | 8.3 Was model estimation conducted appropriately? Were issues of clustering and subgroups handled appropriately? |
| 9. Were the results and conclusions valid? | 9.1 Did study results reflect testable hypotheses and account for statistical uncertainty? |
|  | 9.2 Were study conclusions supported by the evidence and compared with existing findings in the literature? |
|  | 9.3 Were study limitations and generalisability adequately discussed? |
| 10. Was the study presentation clear, concise, and complete? | 10.1 Was study importance and research context adequately motivated? |
|  | 10.2 Were the study data-collection instrument and methods described? |
|  | 10.3 Were the study implications clearly stated and understandable to a wide audience? |

**Table 2: Summary of quality scores for all studies, by item**

| **First author** | **Year** | **Score for each item** | | | | | | | | | | | | | | | | | | | | | | | | | | | | | | **Total score** | **Max score** | **% ^a^** |
| --- | --- | --- | --- | --- | --- | --- | --- | --- | --- | --- | --- | --- | --- | --- | --- | --- | --- | --- | --- | --- | --- | --- | --- | --- | --- | --- | --- | --- | --- | --- | --- | --- | --- | --- |
|  |  | **1.1** | **1.2** | **1.3** | **2.1** | **2.2** | **2.3** | **3.1** | **3.2** | **3.3** | **4.1** | **4.2** | **4.3** | **5.1** | **5.2** | **5.3** | **6.1** | **6.2** | **6.3** | **7.1** | **7.2** | **7.3** | **8.1** | **8.2** | **8.3** | **9.1** | **9.2** | **9.3** | **10.1** | **10.2** | **10.3** |  |  |  |
| Hundley | 2001 | ½ | ½ | ½ | ½ | 0 | 1 | 0 | 0 | 1 | ½ | ½ | 1 |  | ½ | 1 | ½ |  | 1 | 1 | ½ | 1 | ½ | 1 | ½ | ½ | ½ | 1 | ½ | 0 | 1 | 17 | 28 | 61% |
| Hundley | 2004 | 1 | 1 | ½ | ½ | 0 | 1 | 0 | 0 | 1 | 0 | 0 | 1 |  | ½ | 1 | ½ |  | 1 | 1 | ½ | 1 | ½ | 0 | ½ | 1 | ½ | 1 | 1 | 0 | 1 | 17 | 28 | 61% |
| Donaldson | 1998 | ½ | 0 | 1 |  |  |  |  |  |  |  |  |  | 1 |  | 0 | ½ |  | ½ | ½ | 0 | 0 | ½ | ½ |  | 0 | ½ | 1 | ½ | ½ | ½ | 8 | 18 | 44% |
| Longworth | 2001 | 1 | 1 | 1 | 1 | ½ | ½ | 0 | 0 | 1 | ½ | 0 | ½ |  | ½ | 1 | ½ |  |  | 1 | ½ | ½ | ½ | ½ | 1 | 1 | 1 | 0 | 0 | 0 | ½ | 15.5 | 27 | 57% |
| Pitchforth | 2008 | 0.5 | 1 | 0 | 0 | ½ | ½ | 0 | 0 | 1 | 0 | 0 | ½ |  | ½ | 0 | 1 |  |  | ½ | ½ | ½ | 1 | 0 | ½ | 1 | ½ | 1 | 1 | 0 | 1 | 13 | 27 | 48% |
| **% studies meeting criteria** | | 70% | 70% | 60% | 50% | 25% | 75% | 0% | 0% | 100% | 25% | 13% | 75% | 100% | 50% | 60% | 60% | - | 83% | 80% | 40% | 60% | 60% | 40% | 63% | 70% | 60% | 80% | 60% | 10% | 80% | **Mean score** | | **55%** |
| **Ranking of item** | | 9 | 9 | 13 | 20 | 24 | 7 | 28 | 28 | 1 | 24 | 26 | 7 | 1 | 20 | 13 | 13 | - | 3 | 4 | 22 | 13 | 13 | 22 | 12 | 9 | 13 | 4 | 13 | 27 | 4 |  |  |  |

^a^ Studies classified as low quality (fulfilled <50% of applicable quality criteria), average quality (fulfilled between 50% and 80% of applicable criteria) or high quality (fulfilled >80% of applicable criteria)

**Table 3: Summary of quality scores for all studies, by research question**

| **First author** | **Year** | **% of criteria which were fully met** | | | | | | | | | |
| --- | --- | --- | --- | --- | --- | --- | --- | --- | --- | --- | --- |
|  |  | **1** | **2** | **3** | **4** | **5** | **6** | **7** | **8** | **9** | **10** |
| Hundley | 2001 | 50% | 50% | 33% | 67% | 75% | 75% | 83% | 67% | 67% | 50% |
| Hundley | 2004 | 83% | 50% | 33% | 33% | 75% | 75% | 83% | 33% | 83% | 67% |
| Donaldson | 1998 | 50% | - | - | - | 50% | 50% | 17% | 50% | 50% | 50% |
| Longworth | 2001 | 100% | 67% | 33% | 33% | 75% | 50% | 67% | 67% | 67% | 17% |
| Pitchforth | 2008 | 50% | 33% | 33% | 17% | 25% | 100% | 50% | 50% | 83% | 67% |
| **Overall** |  | **67%** | **50%** | **33%** | **38%** | **60%** | **69%** | **60%** | **54%** | **70%** | **50%** |

**References**

1. Donaldson C, Hundley V, Mapp T. Willingness to pay: a method for measuring preferences for maternity care? Birth. 1998;25(1):32-9.

2. Hundley V, Ryan M. Are women's expectations and preferences for intrapartum care affected by the model of care on offer? BJOG : an international journal of obstetrics and gynaecology. 2004;111(6):550-60. doi:10.1111/j.1471-0528.2004.00152.x.

3. Hundley V, Ryan M, Graham W. Assessing women's preferences for intrapartum care. Birth. 2001;28(4):254-63.

4. Longworth L, Ratcliffe J, Boulton M. Investigating women's preferences for intrapartum care: home versus hospital births. Health & social care in the community. 2001;9(6):404-13.

5. Pitchforth E, Watson V, Tucker J, Ryan M, van Teijlingen E, Farmer J et al. Models of intrapartum care and women's trade-offs in remote and rural Scotland: a mixed-methods study. BJOG : an international journal of obstetrics and gynaecology. 2008;115(5):560-9. doi:10.1111/j.1471-0528.2007.01516.x.

6. Bridges JF, Hauber AB, Marshall D, Lloyd A, Prosser LA, Regier DA et al. Conjoint analysis applications in health--a checklist: a report of the ISPOR Good Research Practices for Conjoint Analysis Task Force. Value in health : the journal of the International Society for Pharmacoeconomics and Outcomes Research. 2011;14(4):403-13. doi:10.1016/j.jval.2010.11.013.

7. Abdul Pari AA, Simon J, Wolstenholme J, Geddes JR, Goodwin GM. Economic evaluations in bipolar disorder: a systematic review and critical appraisal. Bipolar Disorders. 2014;16(6):557-82. doi:10.1111/bdi.12213.

8. Ratcliffe J, Longworth L. Investigating the structural reliability of a discrete choice experiment within health technology assessment. International journal of technology assessment in health care. 2002;18(1):139-44.

9. Pitchforth E, van Teijlingen E, Watson V, Tucker J, Kiger A, Ireland J et al. "Choice" and place of delivery: a qualitative study of women in remote and rural Scotland. Quality & safety in health care. 2009;18(1):42-8. doi:10.1136/qshc.2007.023572.

**Appendix: Completed checklist for individual studies**

**1. Hundley 2001**

|  | **Item** | **Score ^a^** | **Comments** |
| --- | --- | --- | --- |
| 1.1 | Were a well-defined research question and a testable hypothesis articulated? | 0.5 | Clear aim stated in Introduction. No testable hypotheses articulated |
| 1.2 | Was the study perspective described, and was the study placed in a particular decision-making or policy context? | 0.5 | The study perspective (decision-making by pregnant women) was described to some degree, and some information about the decision-making context was provided, although this was predominantly covered in the Discussion |
| 1.3 | What is the rationale for using conjoint analysis to answer the research question? | 0.5 | The rationale for using conjoint analysis (a DCE) was partially provided in the Introduction and further comments regarding the rationale were made in the Discussion (e.g. advantages over a simple rating exercise) |
| 2.1 | Was attribute identification supported by evidence (literature reviews, focus groups, or other scientific methods)? | 0.5 | Attribute identification undertaken by reviewing studies of midwife-managed units and policies, and professional recommendations, so was evidence-based but formal strategies were not used |
| 2.2 | Was attribute selection justified and consistent with theory? | 0 | Limited justification provided, and attribute selection was not placed in a theoretical context |
| 2.3 | Was level selection for each attribute justified by the evidence and consistent with the study perspective and hypothesis? | 1 | Yes |
| 3.1 | Was the number of attributes in each conjoint task justified (that is, full or partial profile)? | 0 | No |
| 3.2 | Was the number of profiles in each conjoint task justified? | 0 | No |
| 3.3 | Was (should) an opt-out or a status-quo alternative (be) included? | 1 | An opt-out was not included, but as this is not a decision that women could opt out of making, this design decision was justified |
| 4.1 | Was the choice of experimental design justified? Were alternative experimental designs considered? | 0.5 | Choice of experimental design was justified. Alternative experimental designs were not considered |
| 4.2 | Were the properties of the experimental design evaluated? | 0.5 | The experimental design was evaluated for orthogonality and level balance. Other aspects (e.g. choice probabilities) were not considered |
| 4.3 | Was the number of conjoint tasks included in the data-collection instrument appropriate? | 1 | The number of choice tasks was justified by the authors and was likely to be appropriate |
| 5.1 | Was there sufficient motivation and explanation of conjoint tasks? | N/A | Cannot be determined as the survey instrument was not made available |
| 5.2 | Was an appropriate elicitation format (that is, rating, ranking, or choice) used? Did (should) the elicitation format allow for indifference? | 0.5 | An appropriate elicitation format was used. Indifference was not strictly permitted, but the authors noted instances where indifference led to a choice not being made |
| 5.3 | In addition to preference elicitation, did the conjoint tasks include other qualifying questions (for example, strength of preference, confidence in response, and other methods)? | 1 | Yes. Respondents were also asked about their preferred level for each attribute, and asked to identify their most preferred attribute |
| 6.1 | Was appropriate respondent information collected (such as sociodemographic, attitudinal, health history or status, and treatment experience)? | 0.5 | Appropriate demographic information was collected. However, it was not clear that sufficient attitudinal data were collected |
| 6.2 | Were the attributes and levels defined, and was any contextual information provided? | N/A | Cannot be determined as the survey instrument was not made available |
| 6.3 | Was the level of burden of the data-collection instrument appropriate? Were respondents encouraged and motivated? | 1 | Based on the information provided on response rates, response times and completion rates, for both the pilot and the main survey, the level of burden of the data-collection instrument was likely appropriate |
| 7.1 | Was the sampling strategy justified (for example, sample size, stratification, and recruitment)? | 1 | The sampling strategy was broadly justified, and all key limitations were noted in the Discussion section |
| 7.2 | Was the mode of administration justified and appropriate (for example, face-to-face, pen-and-paper, web-based)? | 0.5 | The mode of administration was likely appropriate, but was not justified |
| 7.3 | Were ethical considerations addressed (for example, recruitment, information and/or consent, compensation)? | 1 | Ethical approval was obtained for the survey |
| 8.1 | Were respondent characteristics examined and tested? | 0.5 | Information on respondent characteristics was presented and discussed, but this information was not incorporated into the regressions that were conducted (e.g. in the form of latent class analysis) |
| 8.2 | Was the quality of the responses examined (for example, rationality, validity, reliability)? | 1 | Yes |
| 8.3 | Was model estimation conducted appropriately? Were issues of clustering and subgroups handled appropriately? | 0.5 | It is likely that an appropriate model was estimated, but limited information was provided on the modelling process |
| 9.1 | Did study results reflect testable hypotheses and account for statistical uncertainty? | 0.5 | The study results did not reflect testable hypotheses, but uncertainty was considered in the form of the significance of model coefficients |
| 9.2 | Were study conclusions supported by the evidence and compared with existing findings in the literature? | 0.5 | The study conclusions were supported by the evidence but were not fully compared with existing findings in the literature |
| 9.3 | Were study limitations and generalisability adequately discussed? | 1 | Yes |
| 10.1 | Was study importance and research context adequately motivated? | 0.5 | This objective was only partially met (which links back to item 1.2) |
| 10.2 | Were the study data-collection instrument and methods described? | 0 | Limited information was provided on the description of attributes and levels and the survey instrument was not made available |
| 10.3 | Were the study implications clearly stated and understandable to a wide audience? | 1 | Yes |
| **Total score (% of max possible)** | | 17/28 (61%) |  |

^a^ 1=fully addressed, 0.5=partially addressed, 0=not addressed

**2. Hundley 2004**

|  | **Item** | **Score ^a^** | **Comments** |
| --- | --- | --- | --- |
| 1.1 | Were a well-defined research question and a testable hypothesis articulated? | 1 | Research question (aim) clearly defined. Hypothesis implicit in the research question |
| 1.2 | Was the study perspective described, and was the study placed in a particular decision-making or policy context? | 1 | The study perspective (decision-making by pregnant women) was described well, and the study was placed in a particular decision-making context |
| 1.3 | What is the rationale for using conjoint analysis to answer the research question? | 0.5 | The rationale for using conjoint analysis (a DCE) was partially provided in the Introduction and Methods sections |
| 2.1 | Was attribute identification supported by evidence (literature reviews, focus groups, or other scientific methods)? | 0.5 | Readers were referred to Hundley 2001 regarding attribute identification, hence the same score is given |
| 2.2 | Was attribute selection justified and consistent with theory? | 0 | Readers were referred to Hundley 2001 regarding the justification of attribute selection, hence the same score is given |
| 2.3 | Was level selection for each attribute justified by the evidence and consistent with the study perspective and hypothesis? | 1 | Readers were referred to Hundley 2001 regarding level selection, hence the same score is given |
| 3.1 | Was the number of attributes in each conjoint task justified (that is, full or partial profile)? | 0 | No |
| 3.2 | Was the number of profiles in each conjoint task justified? | 0 | No |
| 3.3 | Was (should) an opt-out or a status-quo alternative (be) included? | 1 | An opt-out was not included, but as this is not a decision that women could opt out of making, this design decision was justified |
| 4.1 | Was the choice of experimental design justified? Were alternative experimental designs considered? | 0 | Very limited information is given on the experimental design, and readers are not referred to Hundley 2001, hence a lower score is given |
| 4.2 | Were the properties of the experimental design evaluated? | 0 | No |
| 4.3 | Was the number of conjoint tasks included in the data-collection instrument appropriate? | 1 | The number of choice tasks was justified by the authors and was likely to be appropriate |
| 5.1 | Was there sufficient motivation and explanation of conjoint tasks? | N/A | Cannot be determined as the survey instrument was not made available |
| 5.2 | Was an appropriate elicitation format (that is, rating, ranking, or choice) used? Did (should) the elicitation format allow for indifference? | 0.5 | An appropriate elicitation format was used. Indifference was not permitted. This decision was not justified |
| 5.3 | In addition to preference elicitation, did the conjoint tasks include other qualifying questions (for example, strength of preference, confidence in response, and other methods)? | 1 | Yes. Respondents were also asked about their preferred level for each attribute, and asked to identify their most preferred attribute |
| 6.1 | Was appropriate respondent information collected (such as sociodemographic, attitudinal, health history or status, and treatment experience)? | 0.5 | Appropriate demographic information was collected. However, it was not clear that sufficient attitudinal data were collected |
| 6.2 | Were the attributes and levels defined, and was any contextual information provided? | N/A | Cannot be determined as the survey instrument was not made available |
| 6.3 | Was the level of burden of the data-collection instrument appropriate? Were respondents encouraged and motivated? | 1 | Based on the information provided on response rates and completion rates, for both the pilot and the main survey, the level of burden of the data-collection instrument was likely appropriate |
| 7.1 | Was the sampling strategy justified (for example, sample size, stratification, and recruitment)? | 1 | The sampling strategy was fully described, and all key limitations were noted in the Discussion section |
| 7.2 | Was the mode of administration justified and appropriate (for example, face-to-face, pen-and-paper, web-based)? | 0.5 | The mode of administration was likely appropriate, but was not justified |
| 7.3 | Were ethical considerations addressed (for example, recruitment, information and/or consent, compensation)? | 1 | Ethical approval was obtained for the survey |
| 8.1 | Were respondent characteristics examined and tested? | 0.5 | Information on respondent characteristics was presented and discussed, but this information was not incorporated into the regressions that were conducted (e.g. in the form of latent class analysis) |
| 8.2 | Was the quality of the responses examined (for example, rationality, validity, reliability)? | 0 | No |
| 8.3 | Was model estimation conducted appropriately? Were issues of clustering and subgroups handled appropriately? | 0.5 | No information was presented which would enable a judgment to be reached regarding the appropriateness of the model that was estimated, and readers were not referred to Hundley 2001 for this information |
| 9.1 | Did study results reflect testable hypotheses and account for statistical uncertainty? | 1 | Yes |
| 9.2 | Were study conclusions supported by the evidence and compared with existing findings in the literature? | 0.5 | The study conclusions were supported by the evidence but were not fully compared with existing findings in the literature |
| 9.3 | Were study limitations and generalisability adequately discussed? | 1 | Yes |
| 10.1 | Was study importance and research context adequately motivated? | 1 | Yes |
| 10.2 | Were the study data-collection instrument and methods described? | 0 | Limited information was provided on the description of attributes and levels and the survey instrument was not made available |
| 10.3 | Were the study implications clearly stated and understandable to a wide audience? | 1 | Yes |
| **Total score (% of max possible)** | | 17/28 (61%) |  |

^a^ 1=fully addressed, 0.5=partially addressed, 0=not addressed

**3. Donaldson 1998**

|  | **Item** | **Score ^a^** | **Comments** |
| --- | --- | --- | --- |
| 1.1 | Were a well-defined research question and a testable hypothesis articulated? | 0.5 | The research question was well-defined but no testable hypothesis was articulated |
| 1.2 | Was the study perspective described, and was the study placed in a particular decision-making or policy context? | 0 | Very limited information was provided on the study setting, and the decision-making context was not described |
| 1.3 | What is the rationale for using conjoint analysis to answer the research question? ^b^ | 1 | This was described in detail in the introductory section of the paper |
| 2.1 | Was attribute identification supported by evidence (literature reviews, focus groups, or other scientific methods)? | N/A | Not applicable – not a DCE study |
| 2.2 | Was attribute selection justified and consistent with theory? | N/A | Not applicable – not a DCE study |
| 2.3 | Was level selection for each attribute justified by the evidence and consistent with the study perspective and hypothesis? | N/A | Not applicable – not a DCE study |
| 3.1 | Was the number of attributes in each conjoint task justified (that is, full or partial profile)? | N/A | Not applicable – not a DCE study |
| 3.2 | Was the number of profiles in each conjoint task justified? | N/A | Not applicable – not a DCE study |
| 3.3 | Was (should) an opt-out or a status-quo alternative (be) included? | N/A | Not applicable – not a DCE study |
| 4.1 | Was the choice of experimental design justified? Were alternative experimental designs considered? | N/A | Not applicable – not a DCE study |
| 4.2 | Were the properties of the experimental design evaluated? | N/A | Not applicable – not a DCE study |
| 4.3 | Was the number of conjoint tasks included in the data-collection instrument appropriate? | N/A | Not applicable – not a DCE study |
| 5.1 | Was there sufficient motivation and explanation of conjoint tasks? ^b^ | 1 | Yes |
| 5.2 | Was an appropriate elicitation format (that is, rating, ranking, or choice) used? Did (should) the elicitation format allow for indifference? | N/A | Not applicable – not a DCE study |
| 5.3 | In addition to preference elicitation, did the conjoint tasks include other qualifying questions (for example, strength of preference, confidence in response, and other methods)? ^b^ | 0 | No additional qualifying questions were asked alongside the WTP question |
| 6.1 | Was appropriate respondent information collected (such as sociodemographic, attitudinal, health history or status, and treatment experience)? | 0.5 | Some sociodemographic information was collected but this was not presented in any detail, and was not accompanied by information on attitudes, health history or treatment experience |
| 6.2 | Were the attributes and levels defined, and was any contextual information provided? | N/A | Not applicable – not a DCE study |
| 6.3 | Was the level of burden of the data-collection instrument appropriate? Were respondents encouraged and motivated? | 0.5 | The level of burden was likely appropriate (although some elements of the data collection instrument were not presented (e.g. demographic questions). Respondents were somewhat encouraged to respond carefully to the WTP questions |
| 7.1 | Was the sampling strategy justified (for example, sample size, stratification, and recruitment)? | 0.5 | Limited justification was provided |
| 7.2 | Was the mode of administration justified and appropriate (for example, face-to-face, pen-and-paper, web-based)? | 0 | This was not described or justified |
| 7.3 | Were ethical considerations addressed (for example, recruitment, information and/or consent, compensation)? | 0 | No |
| 8.1 | Were respondent characteristics examined and tested? | 0.5 | Respondent characteristics were only examined in a limited manner |
| 8.2 | Was the quality of the responses examined (for example, rationality, validity, reliability)? | 0.5 | Response quality was only examined in a limited manner |
| 8.3 | Was model estimation conducted appropriately? Were issues of clustering and subgroups handled appropriately? | N/A | Not applicable – not a DCE study |
| 9.1 | Did study results reflect testable hypotheses and account for statistical uncertainty? | 0 | No |
| 9.2 | Were study conclusions supported by the evidence and compared with existing findings in the literature? | 0.5 | Study conclusions were supported by the evidence but were only compared with existing evidence in a limited manner |
| 9.3 | Were study limitations and generalisability adequately discussed? | 1 | Yes |
| 10.1 | Was study importance and research context adequately motivated? | 0.5 | In a limited manner |
| 10.2 | Were the study data-collection instrument and methods described? | 0.5 | Only the elements of the data collection instrument related to the WTP questions were described in detail |
| 10.3 | Were the study implications clearly stated and understandable to a wide audience? | 0.5 | The study implications were only considered in a limited manner |
| **Total score (% of max possible)** | | 8/18  (44%) |  |

^a^ 1=fully addressed, 0.5=partially addressed, 0=not addressed; ^b^ These criteria refer specifically to DCE or conjoint analysis studies but are equally applicable to WTP studies (e.g. 1.3: What is the rationale for using a WTP study to answer the research question?). As such, this study is assessed against adapted versions of these items.

**4. Longworth 2001**

|  | **Item** | **Score ^a^** | **Comments** |
| --- | --- | --- | --- |
| 1.1 | Were a well-defined research question and a testable hypothesis articulated? | 1 | Yes, in the introduction and methods sections |
| 1.2 | Was the study perspective described, and was the study placed in a particular decision-making or policy context? | 1 | Yes, in the introduction |
| 1.3 | What is the rationale for using conjoint analysis to answer the research question? | 1 | The rationale is provided at the beginning of the paper |
| 2.1 | Was attribute identification supported by evidence (literature reviews, focus groups, or other scientific methods)? | 1 | Attribute identification was supported by both literature reviews and focus groups |
| 2.2 | Was attribute selection justified and consistent with theory? | 0.5 | Attribute selection was not fully justified |
| 2.3 | Was level selection for each attribute justified by the evidence and consistent with the study perspective and hypothesis? | 0.5 | Levels were likely consistent with the study perspective and hypothesis but were not fully justified |
| 3.1 | Was the number of attributes in each conjoint task justified (that is, full or partial profile)? | 0 | No |
| 3.2 | Was the number of profiles in each conjoint task justified? | 0 | No |
| 3.3 | Was (should) an opt-out or a status-quo alternative (be) included? | 1 | An opt-out was not included, but as this is not a decision that women could opt out of making, this design decision was justified |
| 4.1 | Was the choice of experimental design justified? Were alternative experimental designs considered? | 0.5 | This was only partially justified and alternative designs were not considered |
| 4.2 | Were the properties of the experimental design evaluated? | 0 | No |
| 4.3 | Was the number of conjoint tasks included in the data-collection instrument appropriate? | 0.5 | The number of choice tasks was not justified by the authors but was likely to be appropriate |
| 5.1 | Was there sufficient motivation and explanation of conjoint tasks? | N/A | Cannot be determined as the survey instrument was not made available |
| 5.2 | Was an appropriate elicitation format (that is, rating, ranking, or choice) used? Did (should) the elicitation format allow for indifference? | 0.5 | An appropriate elicitation format was used. Indifference was not permitted. This decision was not justified |
| 5.3 | In addition to preference elicitation, did the conjoint tasks include other qualifying questions (for example, strength of preference, confidence in response, and other methods)? | 1 | Yes |
| 6.1 | Was appropriate respondent information collected (such as sociodemographic, attitudinal, health history or status, and treatment experience)? | 0.5 | Limited respondent information was collected. This was not described fully in the paper |
| 6.2 | Were the attributes and levels defined, and was any contextual information provided? | N/A | Cannot be determined as the survey instrument was not made available |
| 6.3 | Was the level of burden of the data-collection instrument appropriate? Were respondents encouraged and motivated? | N/A | Cannot be determined as the survey instrument was not made available and no information is presented which would permit the level of burden to be inferred |
| 7.1 | Was the sampling strategy justified (for example, sample size, stratification, and recruitment)? | 1 | Yes |
| 7.2 | Was the mode of administration justified and appropriate (for example, face-to-face, pen-and-paper, web-based)? | 0.5 | The mode of administration was likely appropriate but was not justified |
| 7.3 | Were ethical considerations addressed (for example, recruitment, information and/or consent, compensation)? | 0.5 | Ethical approval was granted for the survey but ethical considerations were not addressed in the paper |
| 8.1 | Were respondent characteristics examined and tested? | 0.5 | Respondent characteristics were partially examined |
| 8.2 | Was the quality of the responses examined (for example, rationality, validity, reliability)? | 0.5 | Quality was examined in a limited way by considering dominance |
| 8.3 | Was model estimation conducted appropriately? Were issues of clustering and subgroups handled appropriately? | 1 | Model estimation was likely appropriate, and models were also estimated for likely relevant subgroups |
| 9.1 | Did study results reflect testable hypotheses and account for statistical uncertainty? | 1 | Yes |
| 9.2 | Were study conclusions supported by the evidence and compared with existing findings in the literature? | 1 | Yes |
| 9.3 | Were study limitations and generalisability adequately discussed? | 0 | No |
| 10.1 | Was study importance and research context adequately motivated? | 0 | No |
| 10.2 | Were the study data-collection instrument and methods described? | 0 | Limited information was provided on the description of attributes and levels and the survey instrument was not made available |
| 10.3 | Were the study implications clearly stated and understandable to a wide audience? | 0.5 | These were not fully stated |
| **Total score (% of max possible)** | | 15.5/27  (57%) |  |

^a^ 1=fully addressed, 0.5=partially addressed, 0=not addressed

**5. Pitchforth 2008**

|  | **Item** | **Score ^a^** | **Comments** |
| --- | --- | --- | --- |
| 1.1 | Were a well-defined research question and a testable hypothesis articulated? | 0.5 | Only partial detail was provided about the research question but testable hypotheses were fully articulated |
| 1.2 | Was the study perspective described, and was the study placed in a particular decision-making or policy context? | 1 | Yes |
| 1.3 | What is the rationale for using conjoint analysis to answer the research question? | 0 | The rationale for using a DCE was not provided |
| 2.1 | Was attribute identification supported by evidence (literature reviews, focus groups, or other scientific methods)? | 0 | No |
| 2.2 | Was attribute selection justified and consistent with theory? | 0.5 | Attribute selection was only partially justified but was likely consistent with theory |
| 2.3 | Was level selection for each attribute justified by the evidence and consistent with the study perspective and hypothesis? | 0.5 | Level selection was not justified but was likely consistent with the study perspective and hypothesis |
| 3.1 | Was the number of attributes in each conjoint task justified (that is, full or partial profile)? | 0 | No |
| 3.2 | Was the number of profiles in each conjoint task justified? | 0 | No |
| 3.3 | Was (should) an opt-out or a status-quo alternative (be) included? | 1 | An opt-out was not included, but as this is not a decision that women could opt out of making, this design decision was justified |
| 4.1 | Was the choice of experimental design justified? Were alternative experimental designs considered? | 0 | No |
| 4.2 | Were the properties of the experimental design evaluated? | 0 | No |
| 4.3 | Was the number of conjoint tasks included in the data-collection instrument appropriate? | 0.5 | The number of tasks was likely appropriate but no justification was provided for this decision |
| 5.1 | Was there sufficient motivation and explanation of conjoint tasks? | N/A | Cannot be determined as the survey instrument was not made available |
| 5.2 | Was an appropriate elicitation format (that is, rating, ranking, or choice) used? Did (should) the elicitation format allow for indifference? | 0.5 | An appropriate elicitation format was used. Indifference was not permitted. This decision was not justified |
| 5.3 | In addition to preference elicitation, did the conjoint tasks include other qualifying questions (for example, strength of preference, confidence in response, and other methods)? | 0 | No additional qualifying questions were reported |
| 6.1 | Was appropriate respondent information collected (such as sociodemographic, attitudinal, health history or status, and treatment experience)? | 1 | Yes |
| 6.2 | Were the attributes and levels defined, and was any contextual information provided? | N/A | Cannot be determined as the survey instrument was not made available |
| 6.3 | Was the level of burden of the data-collection instrument appropriate? Were respondents encouraged and motivated? | N/A | Cannot be determined as the survey instrument was not made available and no information is presented which would permit the level of burden to be inferred |
| 7.1 | Was the sampling strategy justified (for example, sample size, stratification, and recruitment)? | 0.5 | The strategy was described fully but only partially justified |
| 7.2 | Was the mode of administration justified and appropriate (for example, face-to-face, pen-and-paper, web-based)? | 0.5 | Mode of administration was likely appropriate but was not justified |
| 7.3 | Were ethical considerations addressed (for example, recruitment, information and/or consent, compensation)? | 0.5 | Ethical approval was not required for the survey but ethical considerations were not addressed in any detail in the paper |
| 8.1 | Were respondent characteristics examined and tested? | 1 | Yes |
| 8.2 | Was the quality of the responses examined (for example, rationality, validity, reliability)? | 0 | No |
| 8.3 | Was model estimation conducted appropriately? Were issues of clustering and subgroups handled appropriately? | 0.5 | Estimation was handled somewhat appropriately. Although subgroup analyses were presented, these used simple methods and more complex regression approaches (which may have been appropriate) were not considered |
| 9.1 | Did study results reflect testable hypotheses and account for statistical uncertainty? | 1 | Yes |
| 9.2 | Were study conclusions supported by the evidence and compared with existing findings in the literature? | 0.5 | Study conclusions were supported by the evidence, but were only compared with existing findings in a limited manner |
| 9.3 | Were study limitations and generalisability adequately discussed? | 1 | Yes |
| 10.1 | Was study importance and research context adequately motivated? | 1 | Yes |
| 10.2 | Were the study data-collection instrument and methods described? | 0 | Limited information was provided on the description of attributes and levels and the survey instrument was not made available |
| 10.3 | Were the study implications clearly stated and understandable to a wide audience? | 1 | Yes |
| **Total score (% of max possible)** | | 13/27  (48%) |  |

^a^ 1=fully addressed, 0.5=partially addressed, 0=not addressed
