# Supplementary material for: Women’s birth place preferences in the United Kingdom: a systematic review and narrative synthesis of the quantitative literature
Source: BMC Pregnancy Childbirth. 2016 Aug 8;16:213. doi: 10.1186/s12884-016-0998-5 (PMC4977690; doi:10.1186/s12884-016-0998-5)
Supplement: Additional file 5: — Narrative summary of findings from each of the included studies. The file provides a table summarising results from each study relating to (a) stated preferences and (b) factors that women report influenced their choice of unit or birth setting. (DOCX 36 kb) [file 12884_2016_998_MOESM5_ESM.docx]

**Additional file 5: Narrative summary of findings from each of the included studies**

| **Report** | **Stated preferences** | **Factors that women report influenced their choice of unit or birth setting** |
| --- | --- | --- |
| Donaldson (1998)  **Stated preference** | When asked to express a preference between the two options (OU vs AMU), 33% of women did not express a preference, 55% expressed a preference for the AMU and 11% for the OU (labour ward).  There appeared to be a possible trend towards women in households falling in lower social class groups being more likely to prefer an OU (25% in women in social class IV or V or unemployed, 12.5% in women in social class III and 6.4% in women in household in social classes I and II). The study also analysed 'willingness to pay' (a method that can be used to assess strength of preference for different options). However, because of a small sample and high level of missing data the findings are difficult to interpret. | Amongst the women (n=57) who expressed a preference for the AMU, 28% commented about it being ‘more homely and relaxed’, 14% ‘more personal’, 12% commented on women being more involved in the unit and 12% commented on continuity of care. Around 10% stated that their preference was not strong. Among the women who preferred the OU (n=11), three mentioned equipment, one mentioned interventions and two made general comments. |
| Emslie (1999)  **Survey** | Women's opinions on the features that they considered important changed between 14 and 36 weeks. The top five at 14 weeks were 'quiet atmosphere' (89%), 'baby with you at all times' (87%), 'availability of specialist facilities' (85%), 'convenience for visitors' (82%) and choices in pain relief '(81%). At 36 weeks 'quiet atmosphere' remained at the top of the list (99%) followed by 'choices in pain relief' (95%), 'availability of specialist facilities' (94%), 'baby with you at all times' (93%) and 'choices in delivery' (91%).  Survey data relating to antenatal care preferences indicated that women rated the importance of seeing the same staff at each antenatal visit highly.  The top five factors that women mentioned at 36 weeks as being important to them with regard to labour were: 'partner being there' (89%), 'availability of specialist staff/equipment' (65%), 'being kept informed' (58%), 'being involved in decisions' (53%) and 'time alone with partner' (42%). Other factors mentioned less frequently included: 'choice of pain relief' (40%), ‘freedom to choose different positions' (30%), 'handed baby immediately' (30%), ‘cared for by known staff’ (28%), ‘not being left alone’ (21%), 'homely atmosphere' (18%), 'care for [sic] by named midwife' (18%). | Factors which women reported influenced their birthplace choice varied according to parity. For nulliparous women the top five reasons at 14 weeks were: ‘distance from home’ (59%), ‘convenience for family’ (51%), ‘reputation of unit/hospital’ (40%), ‘atmosphere of unit/ward’ (35%) and ‘experience of friends’ (30%). ‘Advice of midwife’ was mentioned by 29%, ‘specialist back up’ by 25% and ‘proximity to partners work’ by 24%. For multiparous women the top five reasons were: ‘previous experience’ (75%), ‘distance from home’ (44%), ‘convenience for family’ (44%), ‘specialist back up’ (33%) and ‘reputation of unit/hospital’ (32%). Factors that were significantly more influential for nulliparous women compared with multiparous women were 'experience of friends' and 'advice of midwife'. |
| Hundley (2001)  **Discrete choice experiment** | Of the attributes presented to participants in ‘hypothetical scenarios’ (known midwife, pain relief options, monitoring, appearance of room, medical staff involvement, decision-making style), style of decision-making was reported to be the most important by 40% of women, followed by pain relief (23%), 'known midwife' (17%), involvement of medical staff (13%), type of monitoring (7%) and homely vs clinical appearance of the room (<2%). In the discrete choice experiment (DCE) all attributes significantly influenced women's choices. Regression analyses showed that women tended to prefer scenarios with more continuity of midwife, more pain relief options, continuous monitoring rather than intermittent; and tended to prefer scenarios where medical staff were routinely involved and where women had greater levels of involvement in decision-making. In the DCE using hypothetical scenarios the findings relating to fetal monitoring and involvement of medical staff were in the opposite direction to women’s stated preferences when asked to simply report their preferences by selecting from a list. The authors report that ‘the results of the regression model suggest that respondents preferred maternity units that offered greater continuity of caregiver from the midwife, more methods of pain relief, continuous fetal heart rate monitoring, a homely appearance, routine involvement of medical staff and greater involvement for the woman in the decision-making process.’ (Hundley (2001), p258) |  |
| Hundley (2004)  **Discrete choice experiment** | Women in all three geographical study areas stated that they preferred to have labour care from a midwife that they had met during pregnancy, and to have all methods of pain relief available, intermittent fetal monitoring, a homely environment, involvement of medical staff only if required and greater control/involvement in decision making. In the study area with least continuity available, women were significantly less likely to prefer the option of labour care from a midwife that they had met during pregnancy (52% vs 72-75% in other areas). In the DCE regression analysis, all six attributes (continuity of midwife, pain relief, monitoring, room appearance, medical staff involvement and decision making style) were important to women in the Aberdeen (OU/AMU) area but pain relief was not a significant attribute in the other two (FMU and OU/AMU without epidural) areas. The authors comment that their findings are consistent with an 'endowment effect', that is expectations influence preferences. |  |
| Lavender (2005)  **Survey** | The statements that elicited the strongest agreement related to the presence of a special care baby unit, proximity of antenatal care to home, the importance of being assisted by a midwife at the birth, being willing to travel for higher quality care and feelings of lack of safety if a specially trained doctor was not immediately available:   - ‘It is important to me that a special care baby unit is in the same place that I give birth’: 35% strongly agreed and overall 73% agreed or strongly agreed. - ‘It is important that my antenatal appointments are at a location close to where I live’: 72% agreed or strongly agreed. - ‘It is important to me that a midwife helps me to give birth to my baby even if complications develop’: 69% agreed or strongly agreed. - ‘I would be willing to travel if it meant I would receive higher quality care for my baby and me around the time of birth’: 68% agreed or strongly agreed. - ‘I would feel unsafe if a specially trained doctor was not immediately available when I am in labour’: 62% agreed or strongly agreed.   Although many women felt it important to be helped by a midwife even if complications developed only 20% agreed or strongly agreed that they wanted to ‘be looked after by midwives and not have doctors involved’ and (as noted above) 62% considered that they would feel unsafe if they did not have a ‘specially trained doctor’ immediately available in labour. A statement regarding the importance of care by a "midwife I know" for the baby's birth did not elicit strong responses: few strongly agreed or strongly disagreed with most respondents fairly equally divided between agreeing, disagreeing and neither agreeing nor disagreeing. Half of the participants considered that it was important to be able to have an epidural any time of the day and night. A quarter of participants agreed that the availability of a pool for labour/birth was important to them; while 46% neither agreed nor disagreed that this was important.  Women's views did not differ by age or level of area deprivation. Nulliparous women were significantly more likely than multiparous women to say that they were willing to travel for antenatal care (72% vs. 65%) and were more likely to say that the availability of a pool was important to them (32% vs.19%). (p52) Compared with white European women, ethnic minority women (n=303) were more likely to say that it was important to have antenatal care close to where they lived (81% vs.71%); were significantly more likely to feel unsafe if a doctor was not available (78% vs. 60%); and were more likely to consider it important to have a special care baby unit available where they gave birth (84% vs. 73%). |  |
| Longworth (2001)  **Conjoint analysis** | The conjoint analysis identified three distinct groups: those with a ‘dominant preference for home births', those with a 'dominant preference for hospital births’ and ‘traders’ that is women who changed their preferences based on the attributes of the service. Amongst women with a dominant preference for hospital birth, conjoint analysis indicated that women had a significant preference for higher levels of continuity of carer and more autonomy in decision-making. They also had a significant preference for a hospital location, access to all forms of pain relief and somewhere without the need for transfer in the event of complications. In contrast, only three attributes appeared to be important to women with a dominant preference for home birth: continuity, location and level of autonomy in decision-making. Amongst 'traders' continuity of carer was the only attribute that significantly influenced preferences with higher levels of continuity being preferred. Amongst other attributes, it appeared that a homely environment was more important than access to an epidural, which in turn was more important than decision-making style and the risk of needing to be transferred. The authors note that the package of care ranked first by 'traders' would be ‘a situation in which there is high continuity of carer, the location of delivery is a maternity unit with a home-like environment, there is access to a birthing pool and gas and air for pain relief, the woman has autonomy in decision-making and there is no need to transfer to another location during labour if a problem develops.’ It is notable that 'continuity' (of midwife) was a significant preference across all groups. |  |
| Pitchforth (2008)  **Discrete choice experiment** | In this discrete choice experiment, regression analysis showed that women living in remote and rural areas preferred shorter travel time to access intrapartum care, preferred to deliver in a maternity unit rather than at home and that an OU was the preferred option. The analysis revealed that women were prepared to travel up to 133 minutes from home to receive consultant (OU) care and that they would travel 16 minutes further to receive consultant-led care vs alternatives. Women living in particularly remote areas were willing to travel further.  Women's risk status, type of care at the last birth, and remoteness/rurality of area of residence all influenced women's willingness to travel. Women were more likely to prefer the birth setting that they had recently given birth in (home, FMU, OU). In summary the authors state that women overwhelmingly preferred an institutional delivery to a home birth and that women on average preferred consultant-led care, but drew a travel time threshold at around 2 hours. |  |
| Rennie (1998)  **Survey** | Antenatally the presence of a birth companion was the most highly rated factor (78% of women considered this 'very important'). The proportions of women rating other factors as 'very important’ were: having 'preferences and wishes followed' (46%), 'in control' 42%, 'able to do what you want' (37%),'same midwife in labour' (27%), 'few interventions' (25%), 'not to lose control of behaviour' (24%) and 'known midwife' (21%). Postnatally significantly more women rated having a birth partner as very important (90% vs 78% antenatally); while the proportion of women rating 'known midwife', 'not to lose control of behaviour' and 'preferences and wishes followed' as ‘very important’ declined postnatally. Antenatally a quarter or more of women rated having a 'known midwife' or the 'same midwife in labour' and 'few interventions’ as neither important nor unimportant (‘don't mind’). With regard to access to a midwife 'easy access' rather than ‘all the time’ or 'only when I say' appeared to be the preferred option antenatally. Being given a 'constant flow' of information in labour appeared to be the preference antenatally; postnatally 76% of women rated 'information in labour' as ‘very important’. With regard to pain relief, 'minimum drugs' appeared to be considered more important than either being pain free or having a drug-free labour; while effective pain relief was rated as very important by 64% of women postnatally. Results suggested a preference for shared decision making antenatally; postnatally 46% of women considered it ‘very important’ ‘to have control of decision making’. |  |
| Rogers (2011)  **Survey** |  | Overall 63% of women in this sample of 'AMU users' said that they would give birth in the unit if it became an FMU; 17% said that they would not choose the FMU and 19% were unsure. Multiparous women were more likely to say that they would use the FMU than nulliparous women (71% vs. 57%) but the difference was not statistically significant (p=0.1). Amongst the women who said that they would choose the FMU (n=76), the main reasons were ‘the provision of a homely environment, opportunities for a natural birth, use of water in labour and accessibility’. (Rogers (2011), p238) A previous good experience was more likely to be given as a reason in multiparous women, and the availability of water was significantly more likely to be cited by nulliparous women. Amongst the 21 women who said that they would not choose the birth centre when it became an FMU, the main reasons related to preferring a birth centre on the same site as an OU (81%), 'feel[ing] safer' elsewhere (67%) and concerns about transfer (38%). The women who stated that they would choose the FMU tended to agree with a series of statements about the possible benefits of birth in an FMU: ‘provides women-centred care’, ‘provides a safe alternative to a hospital birth’, ‘provides a more natural alternative to a hospital birth’, ‘has considerable advantages to a hospital birth’, ‘provides a safe alternative to home birth’, ‘has considerable advantages to a home birth’, ‘has a more homely/relaxed atmosphere compared to a hospital birth’ ( Rogers (2011), p239 -240). Around two thirds of the 'AMU users' who would not choose an FMU also agreed with many of the statements about some of the perceived positive attributes of an FMU but were less certain about safety: only 29% agreed that an FMU ‘provides a safe alternative to a hospital birth’ (cf 87% of those who would choose an FMU) and only 67% (cf 90%) agreed that an FMU "provides a safe alternative to home birth". |
